# Antihypertensive constituents in Sanoshashinto

**DOI:** 10.1007/s11418-019-01382-9

**Published:** 2020-01-01

**Authors:** Jianbo Wu, Souichi Nakashima, Marina Shigyo, Mutsumi Yamasaki, Sumire Ikuno, Aoi Morikawa, Shigehiko Takegami, Seikou Nakamura, Atsuko Konishi, Tatsuya Kitade, Hisashi Matsuda

**Affiliations:** 1 Department of Pharmacognosy, Kyoto Pharmaceutical University, Misasagi, Yamashina-ku, Kyoto, 607-8412 Japan; 2 Department of Analytical Chemistry, Kyoto Pharmaceutical University, Misasagi, Yamashina-ku, Kyoto, 607-8414 Japan

**Keywords:** Sanoshashinto, Principal component analysis, Berberine, Baicalin, Vasorelaxant effect, Antihypertensive

## Abstract

It has been reported that Sanoshashinto (SanHuangXieXinTang, 三黃瀉心湯), which is composed of Rhei Rhizoma, Scutellariae Radix, and Coptidis Rhizoma, exhibits vasorelaxant effects in vitro and lowers blood pressure of patients. Based on this discovery, in this study, a mixture containing those three materials and combinations of them were extracted with methanol, and the extracts were fractionated into different parts. Effects of all extracts and fractions on high concentration of potassium chloride (High K^+^)- or noradrenaline (NA)-induced contractions of isolated rat aortic rings or helical strips were examined. Qualitative and quantitative HPLC analyses of the extracts and the fractions revealed that the contents of baicalin and berberine in Sanoshashinto methanol extract (SHXXTM) were higher than those of the other constituents. All pharmacological and HPLC data were analyzed by principal component analysis (PCA) software and the results indicated that baicalin, berberine, palmatine, baicalein, and wogonoside contributed significantly to the pharmacological activity. Furthermore, spontaneously hypertensive rats (SHRs) that were orally given SHXXTM or a baicalin–berberine combination showed significantly reduced increase in the rate of systolic blood pressure (SBP) compared to the control group. These findings suggested that Sanoshashinto has significant vasorelaxant effects in vitro and antihypertensive effects in vivo, and baicalin and berberine, which were the principal constituents of Scutellariae Radix and Coptidis Rhizoma, were the main antihypertensive constituents in Sanoshashinto. It was speculated that baicalin and berberine produced vasorelaxant effects by activating the NO/cGMP pathway and that the BK_Ca_ channel and the DAG/PKC/CPI-17 pathway were also involved.

**Electronic supplementary material:**

The online version of this article (10.1007/s11418-019-01382-9) contains supplementary material, which is available to authorized users.

## Introduction

With the improvement of the standard of living worldwide, more and more people are being diagnosed with hypertension, and hypertension has become a global public health issue. It is estimated that more than 1 billion adults have hypertension and the number is increasing yearly [1]. Hypertension is defined by systolic blood pressure (SBP) ≥ 140 mmHg and/or diastolic blood pressure (DBP) ≥ 90 mmHg, and is calculated by multiplying cardiac output (CO) by systemic vascular resistance (SVR) (BP = CO × SVR). Therefore, an increase in either CO or SVR would result in hypertension [2, 3]. Hypertension is a complex disease that may be caused by many factors, including genetic inheritance, high sodium intake, smoking, and so on [4–6]. Hypertension is also the most important contributor to cardiovascular disease, including ischemic stroke, myocardial infarction, heart failure, and peripheral artery disease [3, 7, 8]. Today, hypertension and cardiovascular risk are usually assessed together to help physicians make the correct diagnosis and prescribe the correct drugs. Although hypertension is very complicated and refractory, many studies have shown that hypertensive patients present with obvious changes in vascular tone, which result in changes in SVR and thus hypertension. Because of this, most hypertension drugs target vascular smooth muscles to lower blood pressure, such as angiotensin-converting enzyme (ACE) inhibitors, angiotensin receptor blockers (ARBs), β-blockers, and calcium channel blockers (CCBs), and these drugs are used singly or in combination. However, with the long-time using of drugs by patients, the side effects of hypertension drugs have gradually become manifest. For example, ACE inhibitors cause cough and angioedema, ARBs also cause slight cough and angioedema sometimes, β-blockers usually cause insomnia, hallucinations, and depression, and CCBs usually cause ankle edema, headache, flushing, and tachycardia [9–11]. Although the side effects are not very serious, researchers have sought to find low side effect drugs and shifted their focus onto compound preparations and traditional medicine, which are expected to produce good vasorelaxant and antihypertensive effects [12–14].

Sanoshashinto, called SanHuangXieXinTang (三黃瀉心湯, SHXXT) in China, originated in the Essential Prescriptions of the Golden Cabinet (金匮要略, Jin Kui Yao Lue). It is composed of three materials: Rhei Rhizoma (DaHuang in Chinese medicine, DH), Scutellariae Radix (HuangQin in Chinese medicine, HQ), and Coptidis Rhizoma (Chinese HuangLian in Chinese medicine, CHL), and has been used to lower body temperature and dissipate body dampness since ancient times. Recently, it was found that SHXXT could relax vascular contractions in vitro and lower blood pressure in patients [15–18]. DH contains rhein, emodin, and sennoside A, and acts by lowering serum cholesterol, improving diabetic nephropathy, and exerting an anti-inflammatory effect [19, 20]. HQ contains baicalin, baicalein, wogonin, and other flavonoids, and produces antihypertensive, anti-inflammatory, and antioxidant effects. Baicalin, the main constituent, induces rat mesenteric arterial relaxation [21, 22]. CHL contains berberine, palmatine, and other alkaloids, and exerts anti-inflammatory, antihypertensive, antihyperglycemic, antiarrhythmic, and antidepressant effects [20, 23]. Berberine, its main constituent, shows good vasorelaxant effects in rat mesenteric artery [24, 25]. Although SHXXT, HQ, and CHL exhibit good antihypertensive and vasorelaxant effects individually, detailed investigations of the antihypertensive and vasorelaxant effects of their combinations in rats, and the possible mechanisms of action are few. Therefore, in this study, a mixture of the three materials and combinations of them were extracted with methanol and fractionated, and all the extracts and the fractions were tested on in vitro antivascular contraction experiments and HPLC analysis. Then, all data were analyzed by PCA software to determine the constituents responsible for vasorelaxant. Furthermore, the extract and the mixture of effective constituents were administered to SHRs to check whether blood pressure was modulated. The possible underlying mechanisms of action in vitro were also discussed.

## Materials and methods

### Plant materials and preparation

The three crude drugs, DH, HQ, and CHL, used in this study, were purchased from Tochimoto Tenkaido Co., Ltd., (Osaka, Japan). The lot numbers of DH, HQ, and CHL used in this study were 007016001, 001116002, and 001317001, respectively.

A blended mixture of DH, HQ, and CHL in 1:1:1 ratio [26] was refluxed with methanol for 1.5 h, and this procedure was repeated three times. The product collected by refluxing was filtered. The filtrate was concentrated under reduced pressure at 40 ℃ to obtain the solid extract (yield: 24.8%).

Meanwhile, for PCA, each material and combinations of two materials (DH and HQ, DHHQ; DH and CHL, DHHL; HQ and CHL, HQHL) were also extracted by the same method and fractionated into water- (W), n-butanol- (Bu), and ethyl acetate- (EA) fractions. Details are described in electronic supplementary materials (ESM-1). All samples were stored at 4 ℃ for the next experiments.

### Animal preparation

All procedures and protocols (Nos: PCOG-17–008 and PCOG-17–010) were approved by the Animal Care and Use Committee of Kyoto Pharmaceutical University. Male Sprague–Dawley rats (200–300 g, 7 weeks), male WKY/Izm rats (250–300 g, 10 weeks), and male SHRs (SHR/Izm, 250–300 g, 10 weeks) were housed under constant temperature and illumination conditions. The rats were allowed access to food and water ad libitum. All rats were purchased from Japan SLC, Inc. (Shizuoka, Japan).

### Chemicals

Baicalin, baicalein, chrysophanic acid, emodin, aloe-emodin, and wogonoside were purchased from Tokyo Chemical Industry Co., Ltd., (Tokyo, Japan). Rhein, wogonin, palmatine chloride, coptisine chloride, and berberine chloride were purchased from FUJIFILM Wako Pure Chemical Corporation (Tokyo, Japan). Sennoside A was purchased from Sigma-Aldrich Co. LLC (Darmstadt, Germany). Calphostin C and *N*^G^-nitro-L-arginine methyl ester hydrochloride (L-NAME) were purchased from Abcam PLC (Cambridge, England). Rottlerin was purchased from Enzo Life Science, Inc. (Farmingdale, USA).

Methanol, acetonitrile, and dimethyl sulfoxide (DMSO) for sample preparation and liquid chromatography were of HPLC grade. All other chemicals and reagents were of analytical reagent grade.

### HPLC analysis

The HPLC system consisted of a Shimadzu LC-20AR Prominence liquid chromatograph pump, an SIL-10AD Prominence autosampler, an SPD-M10A Prominence diode array detector (DAD), a CTO-10ASvp column oven, and an SCL-10A Prominence communications bus module, and data were recorded by LabSolutions software (Version 5.42 SP6) (Shimadzu Co., Kyoto, Japan). Liquid chromatographic separation was achieved using a YMC-Triart-PFP C18 column (250 mm × 4.60 mm, 5 μm) (YMC Co., Ltd., Kyoto, Japan) and column temperature was kept constant at 25 ℃. The mobile phase was composed of a mixture of acetonitrile (A) and water with 0.01 M 1-pentanesulfonic acid sodium salt plus 0.11 mL/L H_3_PO_4_ (B), and was delivered at the flow rate of 1 mL/min. The detection wavelength was set at 270 nm and the loading volume is 10 μL [27]. All the reference standards and the SHXXT samples were dissolved in methanol and made stock solutions for detection. The gradient program is shown in Table 1.

**Table 1:** HPLC gradient program

Time (min)	A: Acetonitrile	B: 0.01 M 1-Pentanesulfonic acid sodium salt plus 0.11 mL/L H_3_PO_4_
0	15	85
30	25	75
50	30	70
75	50	50
90	90	10
100	90	10

The preparative HPLC system consisted of a Shimadzu LC-6AD liquid chromatograph pump, an SPD-10A detector (Shimadzu Co., Kyoto, Japan), and a YMC-Triart-Phenyl C18 column (250 mm × 10 mm, 5 μm) (YMC Co. Ltd., Kyoto, Japan). The mobile phase consisted of a mixture of acetonitrile (A) and water with 0.015 M ammonium formate plus 0.5% formic acid (B) (A:B = 28:72), and the flow rate was 5 mL/min. The detection wavelength was set at 270 nm and the loading volume was 0.3 mL.

### PCA analysis

Thirty-nine peaks were totally detected with different retention times in the HPLC chromatograms of 28 samples. Each peak area of their peaks was calculated for every samples and saved in a single EXCEL to form a 2D data matrix with dimensions of 28 samples (objects) × 39 peaks (variables) (electronic supplementary materials 2, ESM-2). These data were imported to multivariate data analysis “The Unscrambler^®^ X” software (Camo Analytics Co., Oslo, Norway) for PCA. The PCA was carried out after the preprocessing of mean center.

### Tissue preparation

Rat thoracic aorta were carefully removed and cut into 2–3 mm long rings with endothelium (hereinafter “endothelium-intact rings”) or helical strips without endothelium (hereinafter “endothelium-denuded strips”; ca. 2 mm wide × 15 mm long). The ring preparations were set on stainless steel wires and suspended in a 5 mL bath, and the helical strips were placed on stainless steel wires and suspended in individual 6 mL organ baths. All the baths were filled with Krebs solution with the following composition (mM): NaCl 118, KCl 4.8, CaCl_2_ 2.5, MgSO_4_ 1.2, KH_2_PO_4_ 1.2, NaHCO_3_ 24, and D-glucose 11. The solution was maintained at 37 ℃ and aerated with 95% O_2_ plus 5% CO_2_. Contractions of rings were measured with WinDaq Data Acquisition software (Ohio, USA) and those of helical strips were measured isometrically with a force–displacement transducer (ML T0201/D, ADInstruments Pty Ltd., New South Wales, Australia) and recorded using a software, Chart v3.6.8 for PowerLab/MacLab (ADInstruments). A 1-h equilibration period was allotted before initiation of experiments. After equilibration, 2 M High K^+^ (0.18 mL, final concentration 60 mM) and 10^–3^ M NA (5 or 6 μL, final concentration 10^–6^ M) were added to the bath separately. The tissues were washed three times and re-equilibrated after the contractions reached a maximum. This procedure was repeated, the second contraction was obtained, and the test samples were cumulatively added into the bath. In the presence of different activators or inhibitors, all the activators or inhibitors were added before the second contraction and maintained for 15 min and then High K^+^ and NA were added to induce the second contraction. After that, the test samples were cumulatively added into the bath. All SHXXT extracts were dissolved in 100 mg/mL DMSO for in vitro antivascular contraction experiments.

### Blood pressure measurement

SHRs were randomly divided into seven groups where each group had at least six rats. WKY rats were used as the normal group. All rats were housed for 1 week to adapt the environments. The initial average SBP of SHRs used in the experiments was 180 ± 10 mmHg. The doses of the samples for oral administration are described in Table 2. Rat body weights were measured two times a week, and heart rates and blood pressures were measured once a week. SBPs of SHRs were evaluated by a noninvasive tail cuff method using BP-98A (Softron Co., Ltd., Tokyo, Japan).

**Table 2:** Oral administration samples and doses

Group	Dose
Normal (WKY rats)	0.5% CMCNa solution
Control (SHR)	0.5% CMCNa solution
Nifedipine	5 mg/kg/day
SHXXTM-low-dose	200 mg/kg/day
SHXXTM-middle-dose	400 mg/kg/day
SHXXTM-high-dose	800 mg/kg/day
Baicalin and berberine	32 mg + 26 mg/kg/day

### Statistical analysis

Data were expressed as means ± S.E.M. Significant differences between groups were assessed by one-way analysis of variance (1-ANOVA) followed by Dunnett’s method. A P value less than 0.05 was considered significant.

## Results

### HPLC analysis

#### HPLC analysis of SHXXT extracts

**Fig. 1:**
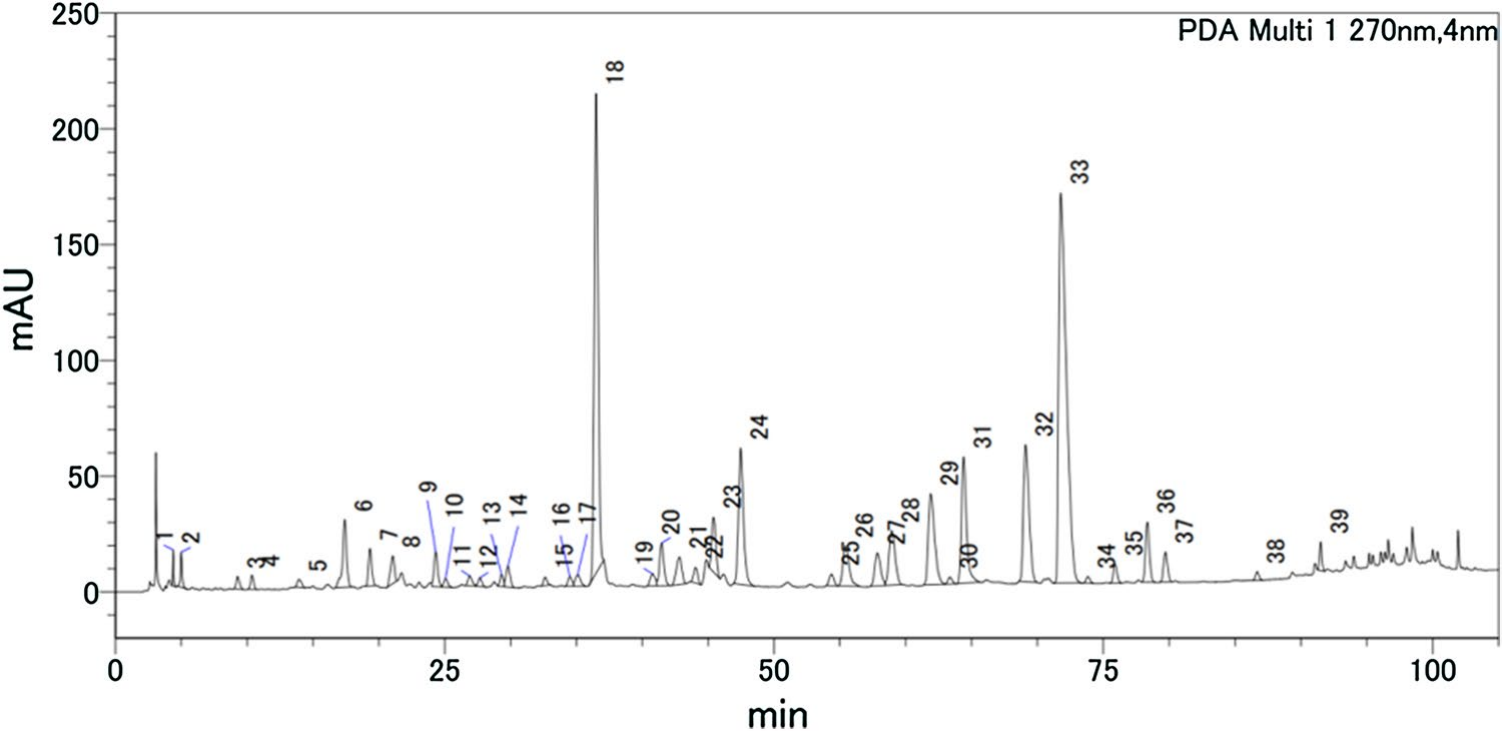
HPLC chromatogram of SHXXTM. The identified peaks were sennoside A (14), baicalin (18), wogonoside (24), coptisine (29), baicalein (31), palmatine (32), berberine (33), rhein (34), wogonin (37), emodin (38), chrysophanic acid (39), respectively

Around 39 peaks were detected in the methanol extract, as indicated in the HPLC chromatogram shown in Fig. 1. We identified 11 compounds in SHXXT methanol extract (SHXXTM) compared with the reference standards, and the amounts of baicalin, berberine, wogonoside, coptisine, baicalein, and palmatine were higher than the other compounds. At the same time, we measured the amounts of baicalin, berberine, baicalein, and palmatine in SHXXTM and the results are shown in Table 3.

**Table 3:** Amounts of baicalin, berberine, baicalein, and palmatine in SHXXTM

Compound	Amount (mg/100 mg)
Baicalin	8.0
Berberine	6.6
Baicalein	2.0
Palmatine	1.8

All samples for PCA were also analyzed by HPLC and the samples were found to have different peak distributions. The extraction procedure and the HPLC data are described in electronic supplementary materials (ESM-1).

#### Preparative HPLC analysis of SHXXTM

**Fig. 2:**
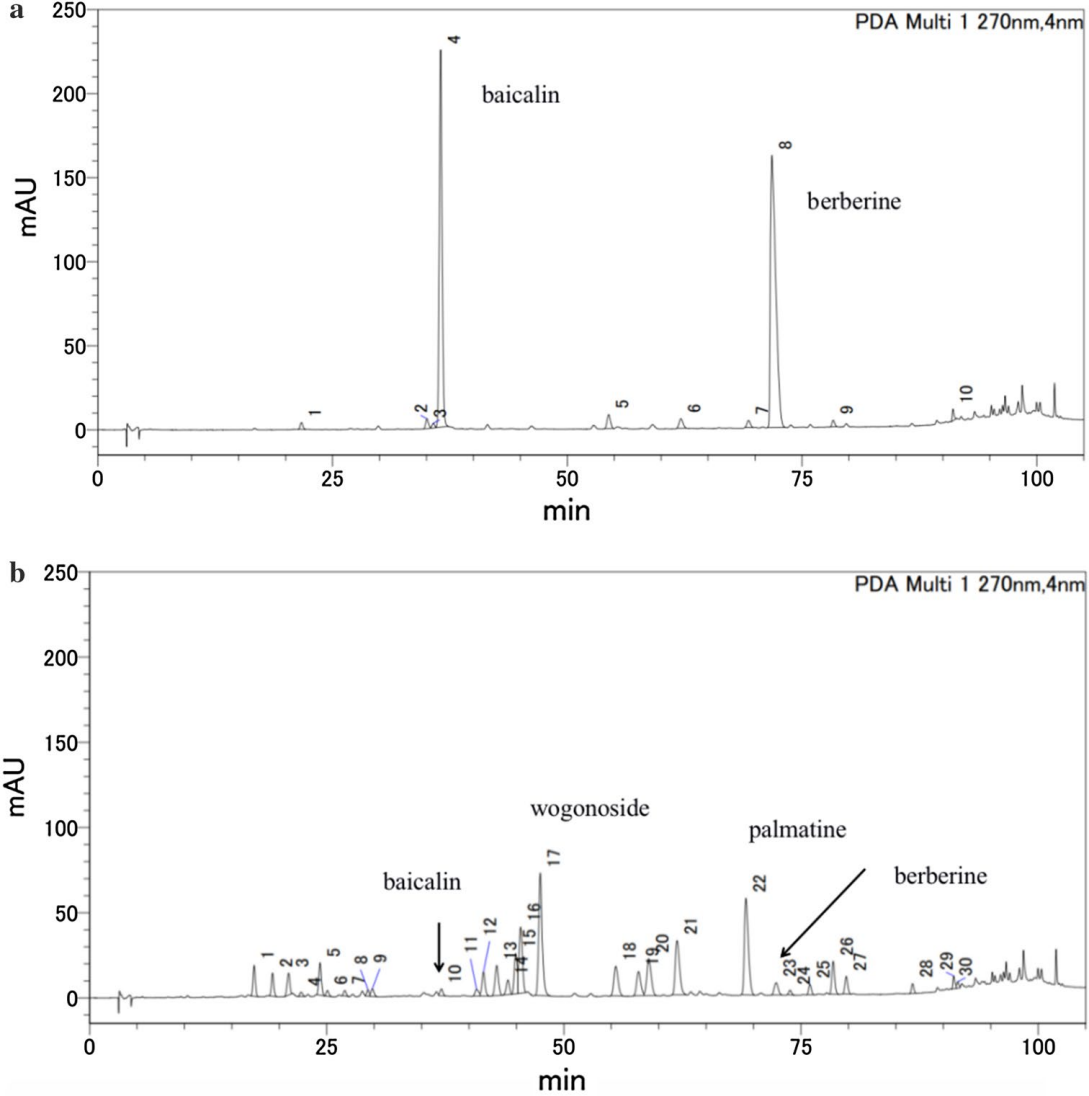
HPLC chromatograms of the two parts of SHXXTM obtained by preparative HPLC. **a** HPLC chromatogram of SHXXTM baicalin and berberine part (SHXXTM–PHPLC–BB). **b** HPLC chromatogram of SHXXTM–except baicalin and berberine parts (SHXXTM–PHPLC–except BB)

As described in “PCA results”, indicated that baicalin, berberine, palmatine, baicalein, and wogonoside markedly contributed to the pharmacological activity. Because the HPLC chromatogram revealed that the amounts of baicalin and berberine were higher than those of the other compounds, we used preparative HPLC to fractionate SHXXTM into the baicalin and berberine part (SHXXTM–PHPLC–BB) and SHXXTM except baicalin and berberine part (SHXXTM–PHPLC–except BB). The chromatograms of the two parts are shown in Fig. 2.

### Effects of SHXXTM on high K^+^- and NA-induced vascular contractions

**Fig. 3:**
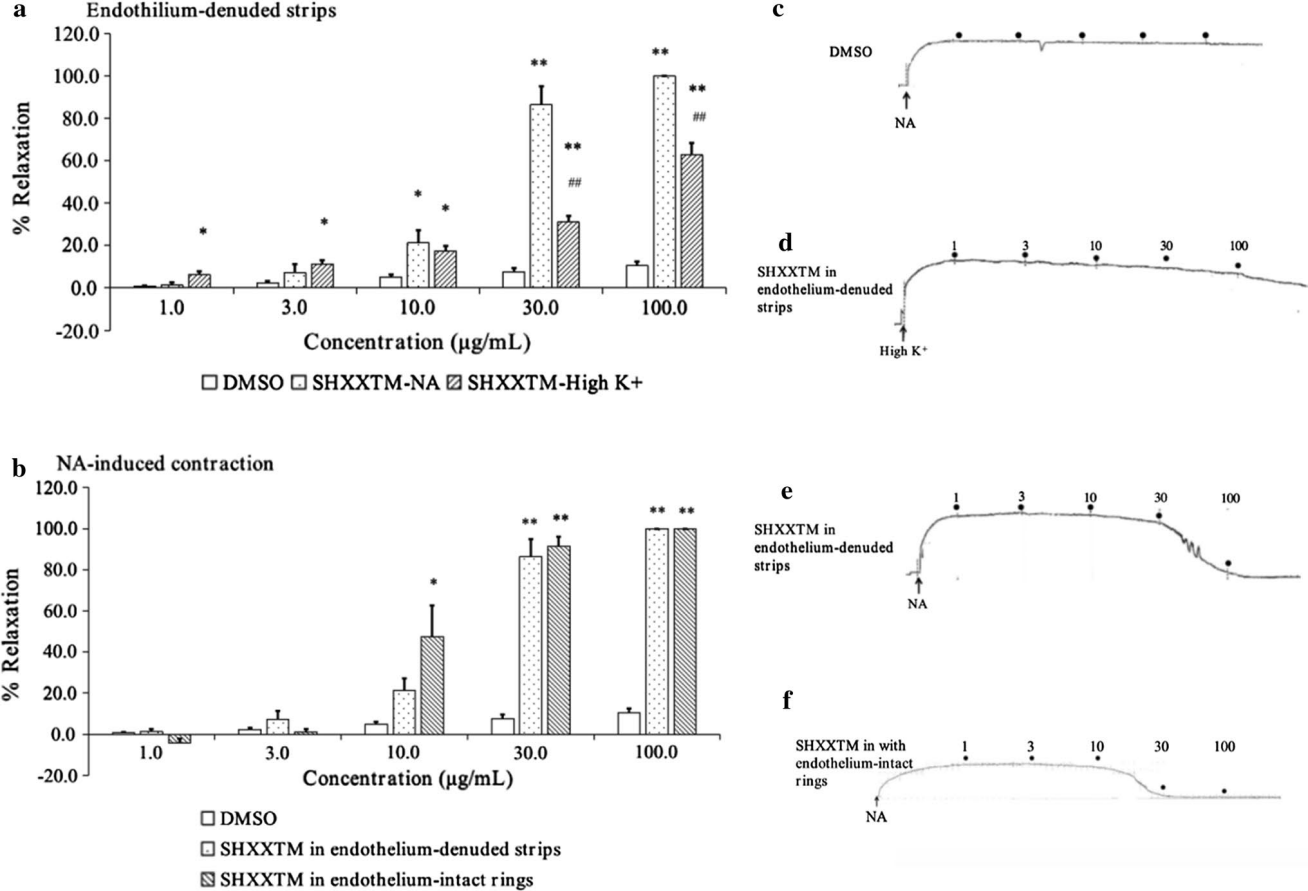
Effects of SHXXTM on High K^+^- and NA-induced contractions in isolated aorta. **a** Relaxation effect of SHXXTM on NA- and High K^+^-induced contractions of endothelium-denuded strips. Each bar graph represents the mean with S.E.M. (*n* = 4–6), *p < 0.05 and **p < 0.01 vs. DMSO control group, and ^##^*p* < 0.01 vs. SHXXTM-NA group. **b** Relaxation effect of SHXXTM on NA-induced contractions of endothelium-denuded strips and endothelium-intact rings. Each bar graph represents the mean with S.E.M. (*n* = 4–6), *p < 0.05 and **p < 0.01 vs. DMSO control group. **c**–**f** Graph of DMSO and SHXXTM in High K^+^- and NA-induced contractions of endothelium-denuded strips and endothelium-intact rings. **c** DMSO in NA-induced contraction of endothelium-denuded strips. **d** SHXXTM in High K^+^-induced contraction of endothelium-denuded strips. **e** SHXXTM in NA-induced contraction of endothelium-denuded strips. **f** SHXXTM in NA-induced contraction of endothelium-intact rings

In isolated aorta, the maximal tension obtained by 60 mM High K^+^ and 10^–6^ M NA was considered to indicate 100% contraction (*n* = 4–6) relative to basal tension. In the endothelium-denuded strips, SHXXTM relaxed the NA-induced vascular contraction with a half maximal effective concentration (EC_50_) of 16.2 ± 1.1 μg/mL, and relaxed the High K^+^-induced vascular contraction with an EC_50_ of 65.1 ± 5.5 μg/mL (Fig. 3a). In the endothelium-intact rings, SHXXTM relaxed the NA-induced vascular contraction with an EC_50_ of 10.5 ± 0.1 μg/mL (Fig. 3b).

### PCA results

PCA was used to clearly visualize the difference of the extracts among 28 samples. A data matrix used was containing 28 objects for which 39 variables have been determined. Usually, PCA calculation could be continued until 39 principal components (PCs) have been obtained. However, in this study, the PCA resulted in a model in which the first two PCs extracted 77% of the total chromatogram variation, the first PC (PC-1) and second PC (PC-2) accounted for 50 and 27% of the total chromatogram variation, respectively. Therefore, the PCA results for PC-1 and PC-2 were considered below.

**Fig. 4:**
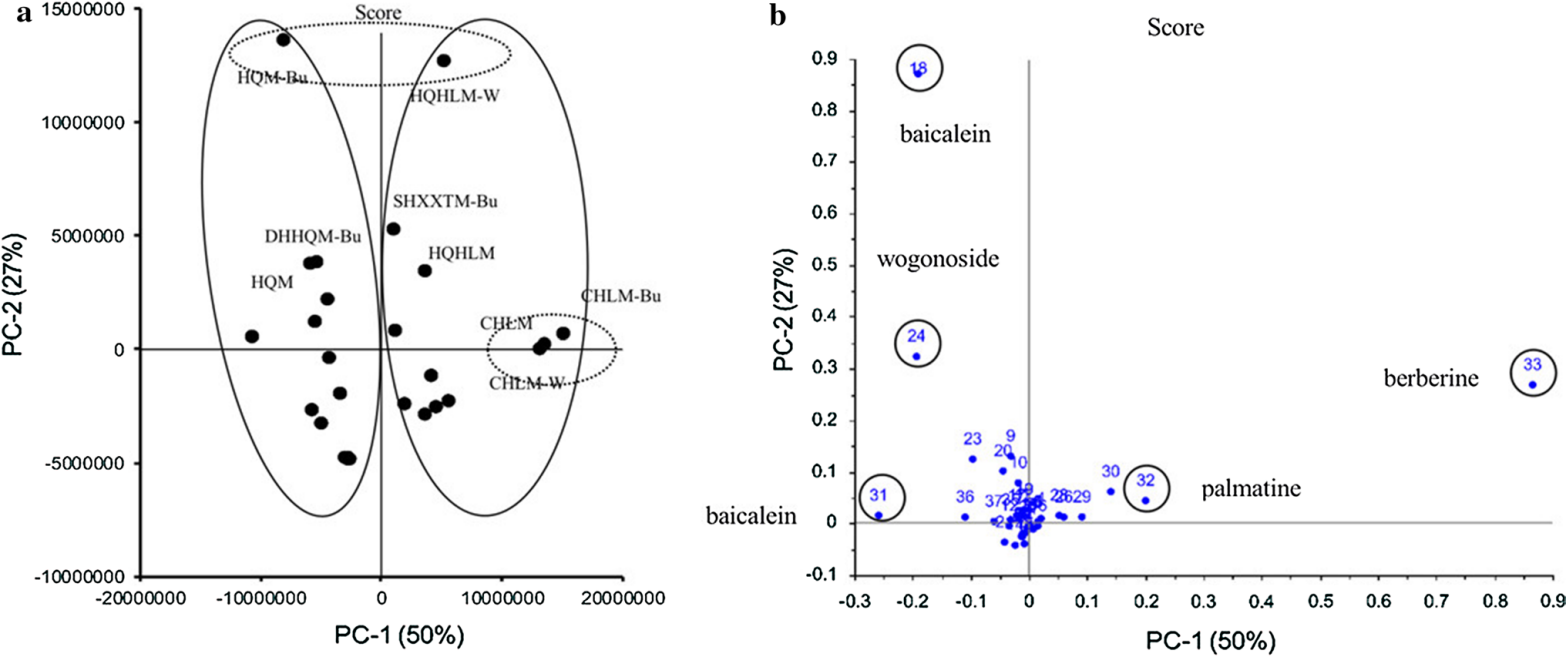
Score and Loading plots of disassembled SHXXT samples by PCA. **a** Score and Loading plots of all samples in PCA results. **b** Score and Loading plots of peaks in PCA results. *SHXXTM* SHXXT methanol extract, *SHXXTM-W* water fraction of SHXXTM, *SHXXTM-Bu* *n*-butanol fraction of SHXXTM, *SHXXTM-EA* ethyl acetate fraction of SHXXTM; the others could refer to SHXXT. (SHXXT—Sanoshashinto, SanHuangXieXinTang in Chinese medicine; DH—Rhei Rhizoma, DaHuang in Chinese medicine; HQ—Scutellariae Radix, HuangQin in Chinese medicine; CHL—Coptidis Rhizoma, Chinese HuangLian in Chinese medicine)

The projections of the points from the original variable spaces on a PC axis are called the Scores of the objects. Based on the PCA calculation, the Score-1 and Score-2 values of all samples were plotted in the two dimensions of PC-1 and PC-2 axis, and are depicted in Fig. 4a. Score plot showed two groups largely apart from the origin (0, 0) on the PC-1 and PC-2 axis. On PC-1 axis, three samples, CHL methanol extract (CHLM), the water fraction of CHLM (CHLM-W), the *n*-butanol fraction of CHLM (CHLM-Bu), had the higher Score-1 values than the other samples. Meanwhile, two samples, HQ methanol extract (HQM-Bu), the water fraction of HQ and HL methanol extract (HQHLM-W), had the higher Score-2 values on the PC-2 axis.

The Score values of the PCs are the weighted sums of the original variables and the weights contain useful information about the variables [28]. These weights are called Loadings and can reveal the variables that are responsible for the main variation in the data. To elucidate the contribution of each original variable to Score-1 and Score-2 values, the Loading plot on the PC-1 and PC-2 axis was depicted in Fig. 4b. On the PC-1 axis, the peak No. 33 (berberine) and No. 32 (palmatine) had the most positive Loading-1 factor, while the peak No. 18 (baicalin), No. 24 (wogonoside) and No. 31 (baicalein) had the negative Loading-1 factors. This indicates that the samples which get the higher Score-1 values in Fig. 4a have higher contents of berberine and palmatine, and lower contents of baicalin, wogonoside and baicalein. In fact, three samples, CHLM, CHLM-W and CHLM-Bu, showed larger peak areas of No. 33 and No. 32 than other samples, and almost no peak No. 18, No. 24, and No. 31 (ESM-1, ESM-2). On the other hand, on the PC-2 axis, although most variables showed positive Loading-2 factors, three of the variables, peak No. 18 (baicalin), No. 24 (wogonoside) and No. 33 (berberine), were comparatively lager in the Loading-2 factor than other variables and the values were in the order of peak No. 18 >  > No. 24 > No. 33. In fact, two samples, HQM-Bu and HQHLM-W, showed larger peak area of peak No. 18 (baicalin) than other samples. However, these two samples had opposite Score-1 values, i.e., HQM-Bu had a negative Score-1 value, while HQHLM-W had a positive Score-1 value, respectively. This is due to that HQHLM-W contained peak No. 33 (berberine) while HQM-Bu did not.

### Effects of baicalin, berberine, palmatine, baicalein, and their combinations on NA-induced contractions of endothelium-denuded strips

**Fig. 5:**
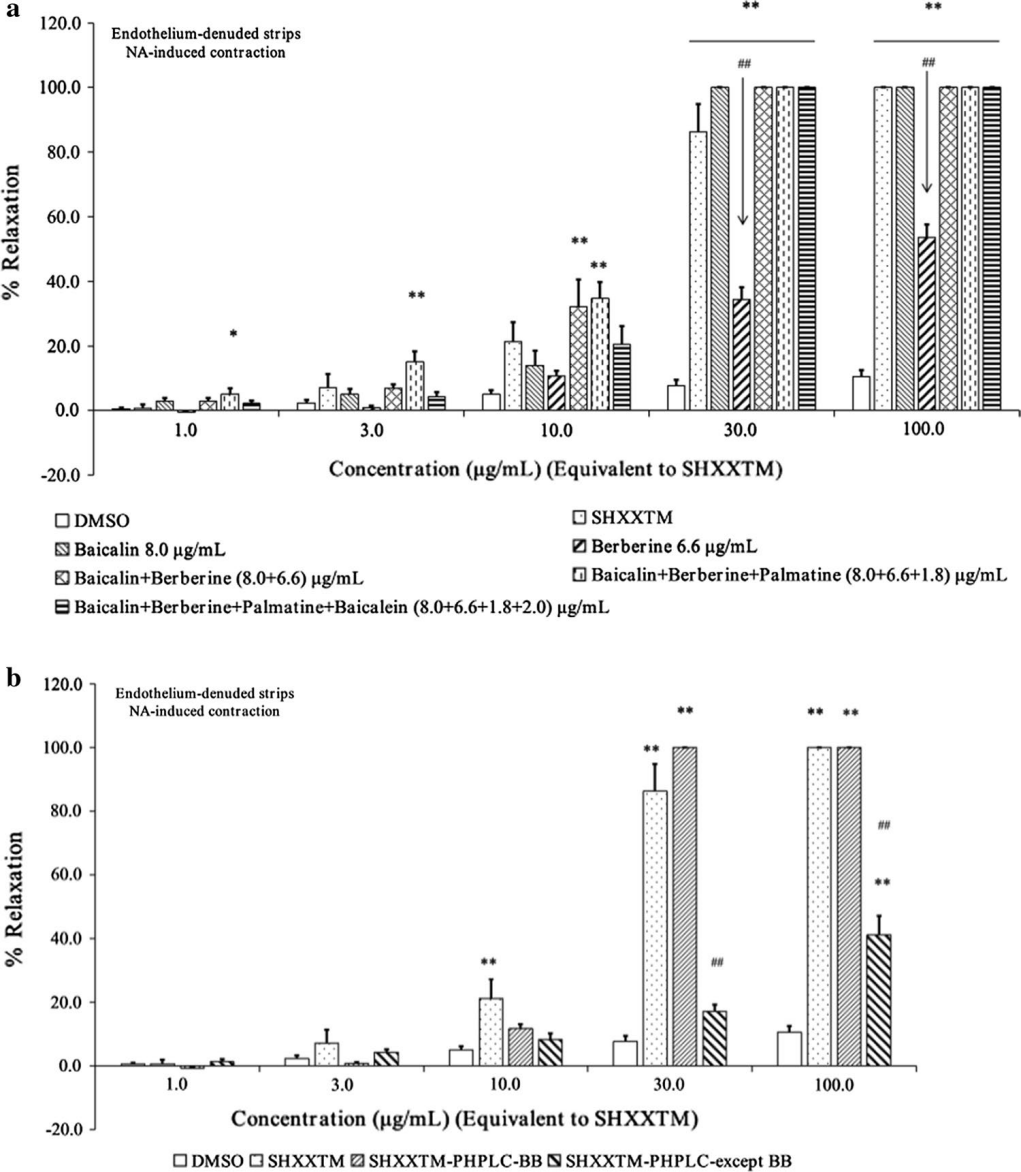
Effects of baicalin, berberine, palmatine, baicalein, and their combinations on NA-induced contractions of endothelium-denuded strips. **a** Vasorelaxant effects of baicalin, berberine, baicalein, palmatine, and their combinations on NA-induced contractions of endothelium-denuded strips. Each graph represents the mean with S.E.M. (*n* = 4–6), *p < 0.05, **p < 0.01 vs. DMSO control group and ^##^*p* < 0.01 vs. SHXXTM group. **b** Vasorelaxant effects of SHXXTM–PHPLC–BB and SHXXTM–PHPLC–except BB on NA-induced contractions of endothelium-denuded strips. Each graph represents the mean with S.E.M. (*n* = 4–6), **p < 0.01 vs. DMSO control group and ^##^*p* < 0.01 vs. SHXXTM group

In the in vitro antivascular contraction experiments, we used SHXXTM at the concentration of 100 mg/mL (final maximum concentration in the medium: 100 μg/mL). As shown in Table 3, HPLC analysis demonstrated that 100 mg of SHXXTM contained 8.0 mg of baicalin, 6.6 mg of berberine, 2.0 mg of baicalein, 1.8 mg of palmatine, and 81.6 mg of the other compounds. Therefore, we used baicalin, berberine, baicalein, and palmatine reference standards and their combinations (the concentrations of them were equal to the concentrations of SHXXTM), to compare and verify the effect in the in vitro experiments. The results are shown in Table 4 and Fig. 5a.

**Table 4:** Vasorelaxant effects of baicalin, berberine, baicalein, palmatine, and their combinations on NA-induced contractions of endothelium-denuded strips

Sample name	Final maximum concentration in medium (μg/mL)				
	1.0	3.0	10.0	30.0	100.0
SHXXTM	0.6 ± 1.2	7.1 ± 4.1	21.2 ± 6.0	86.3 ± 8.6	100.0 ± 0.0
Baicalin 8.0 μg/mL	2.9 ± 0.9	5.0 ± 1.7	13.9 ± 4.5	100.0 ± 0.0	100.0 ± 0.0
Berberine 6.6 μg/mL	0.0 ± 0.3	1.0 ± 0.5	10.7 ± 1.6	34.3 ± 3.8	53.6 ± 4.0
Baicalin + Berberine	2.9 ± 1.0	6.9 ± 1.2	32.1 ± 8.5	100.0 ± 0.0	100.0 ± 0.0
Baicalin + Berberine + Palmatine (8.0 + 6.6 + 1.8) μg/mL	5.1 ± 1.0	15.0 ± 3.2	34.7 ± 5.0	100.0 ± 0.0	100.0 ± 0.0
Baicalin + Berberine + Palmatine + Baicalein (8.0 + 6.6 + 1.8 + 2.0) μg/mL	2.2 ± 0.9	4.3 ± 1.3	20.4 ± 5.8	100.0 ± 0.0	100.0 ± 0.0

Each value represents the mean ± S.E.M. (*n* = 4–6)

The two parts obtained by preparative HPLC were also used in the in vitro experiments for comparsion with SHXXTM, and the results are shown in Fig. 5b. The results indicated that the baicalin and berberine part almost had the same effect compared to SHXXTM.

### Effects of baicalin–berberine combination on NA-induced contractions of endothelium-denuded strips after pretreatment with inhibitors and activators

**Fig. 6:**
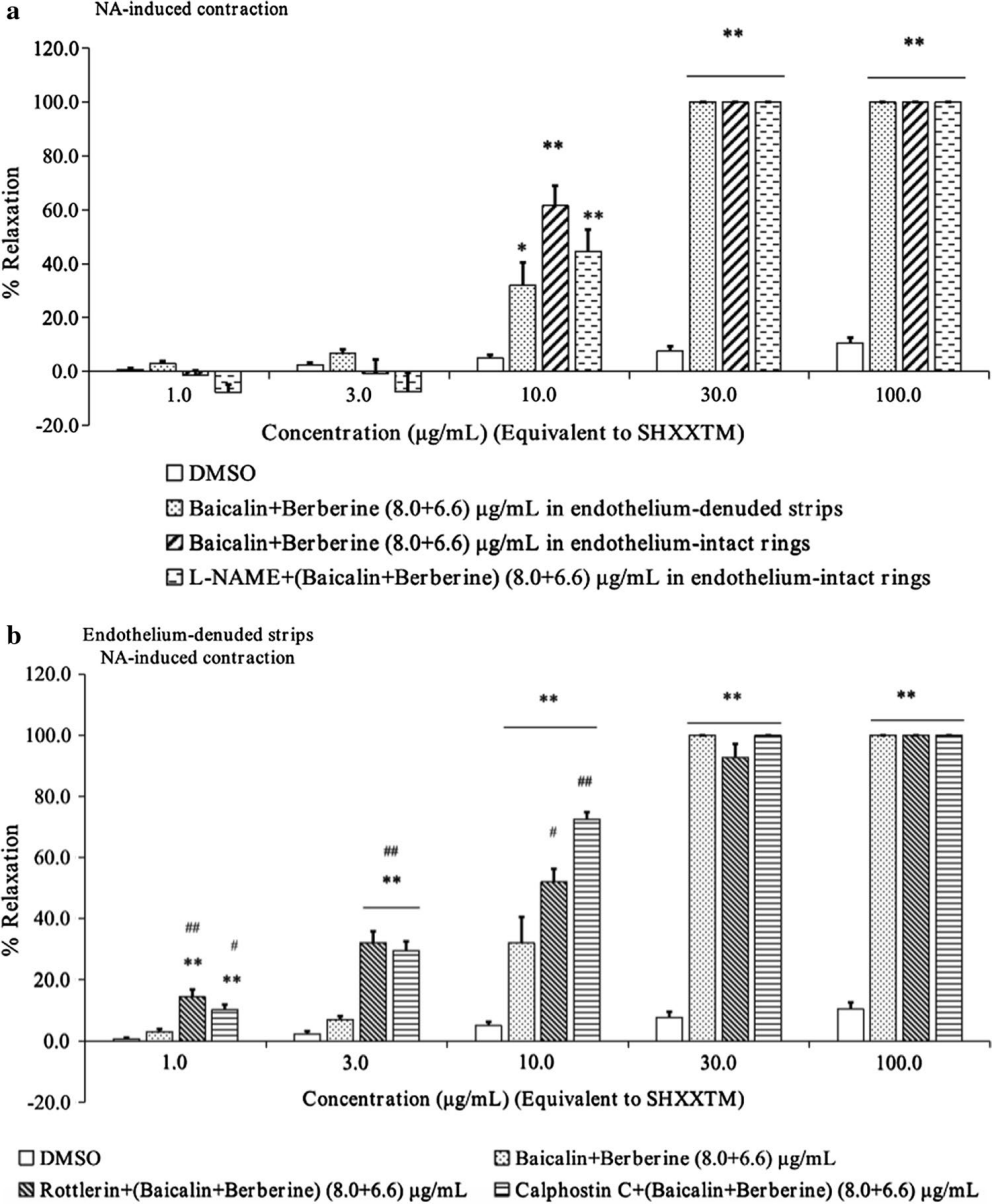
Effects of baicalin–berberine combination on NA-induced vascular contractions after pretreatment with inhibitors and agonists. **a** Vasorelaxant effects of baicalin–berberine combination on NA-induced contractions of endothelium-denuded strips and endothelium-intact rings, and after pretreatment with L-NAME in endothelium-intact rings. Each value represents the mean with S.E.M. (*n* = 4–6), *p < 0.05 and **p < 0.01 vs. DMSO control group. **b** Vasorelaxant effects of baicalin–berberine combination on NA-induced contractions of endothelium-denuded strips after pretreatment with rottlerin and calphostin C. Each bar graph represents the mean with S.E.M. (*n* = 4–6), *p < 0.05 and **p < 0.01 vs. DMSO control group, and ^#^*p* < 0.05 and ^##^*p* < 0.01 vs. baicalin + berberine group

To clarify which channels or pathways are involved in the vasorelaxant effects of SHXXTM, we conducted a preliminary in vitro experiments of a combination of baicalin and berberine. Briefly, we used a nonselective inhibitor of nitric oxide synthetase (NOS) (L-NAME, 1 × 10^–4^ M), a large-conductance Ca^2+^-activated K^+^ (BK_Ca_) channel activator (rottlerin, 3 × 10^–5^ M), and a protein kinase C inhibitor (calphostin C, 2 × 10^–7^ M) for pretreatment of aorta in the in vitro experiments. The results showed that the vasorelaxant effect was increased (10 μg/mL) in endothelium-intact rings, and after pretreated with L-NAME in the rings, the vasorelaxant effect was obviously reduced (10 μg/mL). After pretreatmented with rottlerin and calphostin C in endothelium-denuded strips, the vasorelaxant effect was increased (10 μg/mL), especially calphostin C (Fig. 6).

### Antihypertensive effects of SHXXTM and baicalin–berberine combination in SHRs

After the in vitro study, SHRs were used to verify the antihypertensive effects in vivo. All SHRs were subjected to the procedures described in “Tissue preparation” and the results are shown in Table 5. As shown in Table 5, from the fourth week onward, the SHXXTM-low-dose group, the SHXXTM-middle-dose group, the SHXXTM-high-dose group, and the baicalin–berberine combination group significantly reduced the increase in rate of SBP compared to the control group. In the baicalin–berberine combination group, of which the baicalin–berberine contents were equivalent to the SHXXTM-middle-dose group, almost the same effects were observed as compared to those of the SHXXTM-middle-dose group.

**Table 5:** Effects of various doses of SHXXTM and baicalin–berberine combination on SHRs systolic pressure

Group	Systolic pressure (mmHg) (mean ± S.E.M.)						
	11 weeks (start)	12 weeks	13 weeks	14 weeks	15 weeks	16 weeks	17 weeks
Normal	114.6 ± 2.2**	121.7 ± 2.9**	125.1 ± 2.9**	122.7 ± 3.4**	127.0 ± 2.0**	126.5 ± 2.3**	126.6 ± 2.6**
Control	179.0 ± 1.8	183.7 ± 2.2	184.7 ± 1.4	193.0 ± 2.8	199.4 ± 1.5	204.4 ± 1.5	210.6 ± 1.8
Positive control (Nifedipine)	178.3 ± 1.1	144.3 ± 5.3**	138.4 ± 1.4**	142.4 ± 4.1**	144.2 ± 5.0**	130.1 ± 4.1**	133.1 ± 3.1**
SHXXTM-low-dose	176.5 ± 1.9	176.2 ± 2.4	183.3 ± 3.2	187.5 ± 2.1	188.4 ± 0.8*	192.4 ± 1.2**	195.3 ± 2.0**
SHXXTM-middle-dose	181.3 ± 2.5	180.1 ± 1.8	179.8 ± 1.8	184.9 ± 2.4	186.0 ± 1.9**	187.1 ± 2.0**	190.0 ± 1.4**
SHXXTM-high-dose	176.2 ± 0.9	183.1 ± 5.1	179.6 ± 2.3	182.2 ± 3.7	184.0 ± 3.7**	181.0 ± 2.2**	179.0 ± 1.5**
Baicalin and berberine	180.8 ± 2.1	175.0 ± 1.7	178.0 ± 1.6	185.0 ± 1.4	185.9 ± 1.0**	190.4 ± 2.2**	190.7 ± 2.5**

Each value represents the mean ± S.E.M. (*n* = 6–10). Asterisks denote significant difference from control group, *p < 0.05, **p < 0.01

## Discussion

SHXXT, which is a classical prescription and very famous both in China and Japan. It was recorded that this prescription was suitable for Yo pattern (陽証) and excess pattern (実証), improving the habitus, relieving dizziness, facial redness, nervous anxiety and constipation, also it could ameliorate the symptoms caused by hypertension, such as dizziness, periarthritis of shoulder, tinnitus, insomnia, anxiety and so on. Besides these, it could be used in hemorrhage, for example, nose bleed and hemorrhoid bleed. In addition, Sanogan (三黄丸) and Sanousan (三黄散) composed of the same three materials have been clinically used for the same symptoms in Japan.

In our pre-examination, we compared HPLC profiles and EC_50_ values in vitro experiments of the methanol extract of SHXXT (SHXXTM) and the water extract of SHXXT (SHXXTW). From the results, we found that the number of peaks and their amounts in SHXXTW were decreased except for baicalin. This indicated that all the peaks in SHXXTW were contained in SHXXTM (Figs. S25, S26 and Table S3, ESM-1), but the profile of SHXXTM was observed more peaks than that of SHXXTW, because methanol could extract more liposoluble constituents than water. Meanwhile, we found that the content of berberine in SHXXTW was decreased, because the lots of tannin in DH could combine with berberine and produce water-insoluble complex [29]. In the in vitro experiments, the EC_50_ values of SHXXTM (EC_50_ = 16.2 µg/mL) and SHXXTW (EC_50_ = 11.6 µg/mL) were not so different (Table S4, Fig. S27, ESM-1). Besides these reasons, various data were needed for the PCA to observe the relationship between the peaks, we, therefore, selected methanol as the extraction solvent. But in future, we would like to continue to do research about the SHXXTW, especially the in vivo study.

In Japan, there are other kampo prescriptions which called Gon-Ren Zai (芩連剤), including both of Scutellariae Radix and Coptidis Rhizoma. It has been used for relieving the epigastric distention and pain together with relieving dizziness, facial redness, nervous anxiety, nose bleed and so on. The symptoms such as dizziness, facial redness, nervous anxiety, nose bleed have been considered to accompany with hypertension, but the effects of SHXXT on these symptoms was well as the epigastric distention and pain could not be evaluated in this study.

PCA is a linear dimensionality reduction technique for extracting information from a high-dimensional space by projecting it into a low-dimensional sub-space. PCA preserves essential parts which have more data variation and removes non-essential parts which have less data variation. PCA is commonly used in the analysis of chromatographic data of drugs [30–33]. Traditional prescriptions and medicines usually contain a lot of compounds, which make it difficult for researchers to carry out quality control and elucidate the underlying mechanism. For this reason, many researchers would like to use some methods such as prescription disassembling, HPLC fingerprint, prescription ingredients combination, in vitro pharmacological experiments, in vivo metabolite experiments to take detailed research about them. We also adopted these methods in this study. Briefly, SHXXTM was fractionated and all fractions were analyzed by HPLC, then evaluated by in vitro antivascular contraction experiments. Thereafter, all pharmacological and HPLC data were analyzed by PCA software, and PCA results showed that baicalin, berberine, palmatine, baicalein and wogonoside contributed significantly to the vasorelaxant effect. Two samples, HQM-Bu and HQHLM-W, had higher Score-2 value in PCA calculation showed lower EC_50_ values of 5.2 and 5.0 μg/mL, respectively, than other samples (Table S2). Since HQM-Bu did not contain berberine, it was indicated that baicalin in two samples had high contribution to the pharmacological activity. The n-butanol fraction of SHXXTM (SHXXTM-Bu) and HQHL methanol extract (HQHLM) also showed comparatively low EC_50_ values of 6.7 and 6.9 μg/mL, respectively, although their values were higher than those of HQM-Bu and HQHLM-W. These samples contained baicalin and berberine more rich than other samples except HQM-Bu and HQHLM-W. Meanwhile, HQ methanol extract (HQM) and the n-butanol fraction of DHHQ methanol extract (DHHQM-Bu) had almost same Score-2 value with SHXXTM-Bu and HQHLM. The two samples had EC_50_ values of 15.8 (HQM) and 17.0 μg/mL (DHHQM-Bu) (Table S2), respectively, and showed lower potency than SHXXTM-Bu and HQHLM. Comparing with SHXXTM-Bu and HQHLM sample, HQM and DHHQM-Bu contained the almost same baicalin content and higher wogonoside but did not contain berberine. Therefore, this result indicated that berberine also contributed to the pharmacological activity, although its potency was weaker than that of baicalin. Meanwhile, three samples, CHLM, CHLM-W and CHLM-Bu, with high Score-1 value, and EC_50_ values between two samples, SHXXTM-Bu and HQHLM, and two samples, HQM and DHHQM-Bu, e.g., 8.3 (CHLM-W), 10.8 (CHLM) and 11.0 μg/mL (CHLM-Bu), respectively (Table S2). These samples contained berberine more rich than the other samples. Therefore, this result supported the above consideration that berberine also contributed to the pharmacological activity. In addition, three samples were also high palmatine contents. Therefore, palmatine also may contribute to the pharmacological activity with berberine. On the other hand, the samples localized around the origin (0, 0) in Score plot showed low or no potency to the pharmacological activity. Connected all the HPLC and EC_50_ data, we used principal component regression (PCR) analysis to calculate whether the results were reliable or not. The results showed that the values of predicated EC_50_ and experimentally observed EC_50_ were almost same, and regression coefficients was larger than 0.99 (Fig. S28, ESM-1). To confirm the PCA results, we used preparative HPLC to separate SHXXTM into SHXXTM–PHPLC–BB and SHXXTM–PHPLC–except BB parts, and these two parts were also analyzed by HPLC and tested by in vitro antivascular contraction experiments. From the in vitro results (Fig. 5b), we suggested that SHXXTM–PHPLC–BB had almost the same effect on in vitro antivascular contraction as SHXXTM, whereas the vasorelaxant effects of SHXXTM–PHPLC–except BB were obviously decreased. The results confirmed that the process from prescription disassembling to PCA analysis is suitable for prescription research, and PCA is an accurate and reliable tool for scientific research of traditional medicines.

According to the above results, we selected baicalin and berberine as a combination for more detailed research. In vitro antivascular contraction experiments indicated that the baicalin–berberine combination had almost the same effects as SHXXTM, whereas the application of only baicalin or berberine produced a weak effect compared to SHXXTM, and this result was consistent with PCA results. After the preliminary experiments in vitro study, we proceeded to perform an in vivo study to confirm the antihypertensive effects. From the results in Table 5, we could see that from the fourth week onward, compared to the control group, the SHXXTM-low-dose group, the SHXXTM-middle-dose group, the SHXXTM-high-dose group, and the baicalin–berberine combination group significantly reduced increase in the rate of SBP increase. The results indicated that the SHXXTM groups and the baicalin–berberine combination exhibited a significant antihypertensive effects in vivo. This was consistent with the in vitro study, and it also suggested that SHXXT might be useful in the clinical treatment in future.

**Fig. 7:**
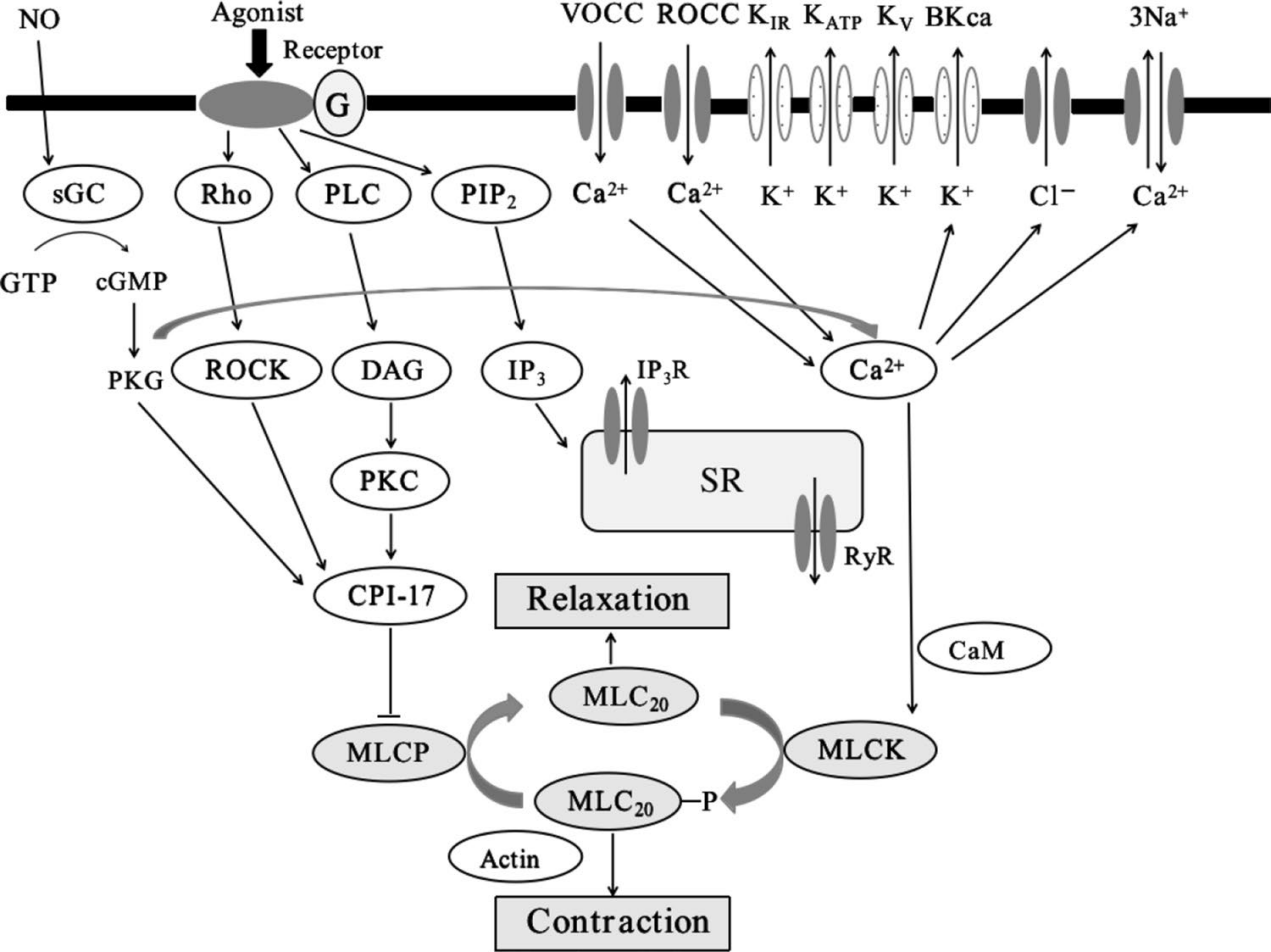
Signaling pathway for vascular smooth muscle contraction. *VOCC* voltage-operated calcium channel, *ROCC* receptor-operated calcium channel, *KIR* inward rectifier potassium channel, *KATP* ATP-sensitive potassium channel, *KV* voltage-gated K^+^ channel, *BKCa* Ca^2+^-activated K^+^ channel, *sGC* soluble guanylyl cyclase, *Rho* Rho-associated kinase, *PLC* phospholipase C, *PIP2* phosphatidylinositol (4,5)-bisphosphate, *GTP* guanosine triphosphate, *cGMP* cyclic guanosine monophosphate, *PKG* protein kinase G, *ROCK* Rho-associated protein kinase, *DAG* diacylglycerol, *IP3* inositol trisphosphate, *SR* sarcoplasmic reticulum, *PKC* protein kinase C, *RyR* ryanodine receptor, *CPI-17* C-kinase potentiated protein phosphatase-1 inhibitor of 17 kDa, *CaM* calmodulin, *MLCK* myosin light chain kinase, *MLCP* myosin light chain phosphatase, *MLC20* 20 kDa myosin light chain

To understand the mechanism of SHXXTM underlying in vasorelaxant effects, we preliminarily used some blockers or activators in the in vitro antivascular experiments to estimate whether the vasorelaxant effects would be changed. From Fig. 3b and Fig. 6a, we found that when we used endothelium-intact rings, the vasorelaxant effects of SHXXTM and the baicalin–berberine combination were enhanced, but after pretreatment with the NOS inhibitor L-NAME, the vasorelaxant effects were reduced. These results suggested that the endothelium was involved in the vasorelaxant effects. Nitric oxide (NO), which is released by endothelial cells, diffuses into vascular smooth muscle cells where it will activate soluble guanylate cyclase (sGC), which in turn catalyzes the production of cyclic guanosine monophosphate (cGMP) from guanosine triphosphate (GTP), then, cGMP induces vascular smooth muscle relaxation by activating cGMP-dependent protein kinase G (PKG). One study showed that cGMP/PKG phosphorylates BK_Ca_ subunit to activate the BK_Ca_ channel, which induces vascular smooth muscle membrane hyperpolarization and subsequently causes vasorelaxant [34]. Therefore, we speculate that baicalin and berberine would promote NO release from the endothelium to activate the NO/cGMP pathway, thereby inducing vasorelaxant. On the other hand, PKG was reported to directly decrease intracellular Ca^2+^ increase, which contributed to vascular contraction [35], although more evidence is needed to verify this. With regard to the BK_Ca_ channel, studies have suggested that high blood pressure and vascular dysfunction are involved in cellular signaling cascades that alter arterial BK_Ca_ channel expression to modify vascular tone further [36, 37]. In this study, we also used rottlerin, a BK_Ca_ channel opener [38], for vessel pretreatment in the in vitro experiment, and the results showed that the relaxation effect was obviously enhanced (Fig. 6b). This result suggested that the BK_Ca_ channel would be involved in the vasorelaxant effects of SHXXTM, consistent with previous findings [21, 24]. Meanwhile, from Fig. 6b, we found that after pretreatment with calphostin C, a protein kinase C (PKC) inhibitor, the vasorelaxant effects were also enhanced, and the enhancement was greater than that by rottlerin. This result indicated that the DAG/PKC/CPI-17 pathway might be involved in the Vasorelaxant effects. C-kinase potentiated protein phosphatase-1 inhibitor of 17 kDa (CPI-17), which dephosphorylates myosin light chain phosphatase (MLCP) into MLC_20_, causes vasorelaxant. Recently, several studies have demonstrated that CPI-17 plays important roles in vascular smooth muscle function [39–42]. The signaling pathways for vascular contraction are shown in Fig. 7 [43, 44]. As described above, the NO/cGMP pathway, the BK_Ca_ channel, and the DAG/PKC/CPI-17 pathway are speculated to be involved.

## Conclusions

In this study, we provided the first piece of evidence that baicalin and berberine are the main effective constituents in SHXXT, because they exhibited the same antihypertensive effects as SHXXT, and the baicalin–berberine combination may replace SHXXT for use as an antihypertensive in the future. We also carried out preliminary experiments to understand the mechanisms of how the baicalin–berberine combination produced vasorelaxant effect, although more experiments are warranted.

## Electronic supplementary material

Below is the link to the electronic supplementary material.

Supplementary file1 (DOCX 2041 kb)

Supplementary file2 (PDF 33173 kb)
