# Correction to: Antihypertensive constituents in Sanoshashinto

**DOI:** 10.1007/s11418-021-01500-6

**Published:** 2021-03-06

**Authors:** Jianbo Wu, Souichi Nakashima, Marina Shigyo, Mutsumi Yamasaki, Sumire Ikuno, Aoi Morikawa, Shigehiko Takegami, Seikou Nakamura, Atsuko Konishi, Tatsuya Kitade, Hisashi Matsuda

**Affiliations:** 1 Department of Pharmacognosy, Kyoto Pharmaceutical University, Misasagi, Yamashina-ku, Kyoto, 607-8412 Japan; 2 Department of Analytical Chemistry, Kyoto Pharmaceutical University, Misasagi, Yamashina-ku, Kyoto, 607-8414 Japan

## Correction to; Journal of Natural Medicines (2020) 74:421–433 10.1007/s11418-019-01382-9

The article Antihypertensive constituents in Sanoshashinto, written by Jianbo Wu, Souichi Nakashima, Marina Shigyo, Mutsumi Yamasaki, Sumire Ikuno, Aoi Morikawa, Shigehiko Takegami, Seikou Nakamura, Atsuko Konishi, Tatsuya Kitade and Hisashi Matsuda, was originally published Online First without Open Access. After publication in volume 74, issue 2, page 421–433 the author decided to opt for Open Choice and to make the article an Open Access publication. Therefore, the copyright of the article has been changed to © The Author(s) 2021 and the article is forthwith distributed under the terms of the Creative Commons Attribution 4.0 International License (https://creativecommons.org/licenses/by/4.0/), which permits use, sharing, adaptation, distribution and reproduction in any medium or format, as long as you give appropriate credit to the original author(s) and the source, provide a link to the Creative Commons license, and indicate if changes were made.

The original article has been updated.

