# Supplementary material for: Antihypertensive constituents in Sanoshashinto
Source: J Nat Med. 2020 Jan 1;74(2):421–33. doi: 10.1007/s11418-019-01382-9 (PMC7929964; doi:10.1007/s11418-019-01382-9)
Supplement: Supplementary file 1 — Supplementary file1 (DOCX 2041 kb) [file 11418_2019_1382_MOESM1_ESM.docx]

**Electric Supplementary Material：**

**1. Disassembled Sanoshashinto prescription extraction**

**1.1 Extraction procedure**

The three crude drugs used in this study, Rhei Rhizoma (DH), Scutellariae Radix (HQ), Coptidis Rhizoma (CHL) were purchased from Tochimoto Tenkaido Co., Ltd. (Osaka, Japan).

A blended mixture of DH, HQ, and CHL in 1:1:1 ratio was refluxed with methanol for 1.5 h, and this procedure was repeated three times. The product collected by refluxing was filtered. The filtrate was concentrated under reduced pressure at 40 ℃ to obtain the solid extract (yield: 24.8%). Besides the whole prescription, for PCA analysis, we also extracted each material and combined materials [Rhei Rhizoma, Scutellariae Radix, Rhei Rhizoma, Rhei Rhizoma and Scutellariae Radix group (1:1), Rhei Rhizoma and Coptidis Rhizoma group (1:1), Scutellariae Radix and Coptidis Rhizoma group (1:1)]. All of them were refluxed with methanol for 1.5 h, and this procedure was repeated three times. The product collected by refluxing was filtered. The filtrate was concentrated under reduced pressure at 40 ℃ to obtain the solid extract. Meanwhile, each sample was fractionated into ethyl acetate-, n-butanol-, and water-soluble fractions. All samples were stored at 4 ℃ for the next experiments.

**Table S1** The yields of the methanolic extracts

| **Extracts name** | **yield%** |
| --- | --- |
| SHXXT | 24.8 |
| DH | 35.6 |
| HQ | 28.3 |
| CHL | 16.9 |
| HQ and CHL | 25.8 |
| DH and CHL | 22.9 |
| DH and HQ | 31.7 |

**1.2 HPLC profiles of the extracts and fractions**


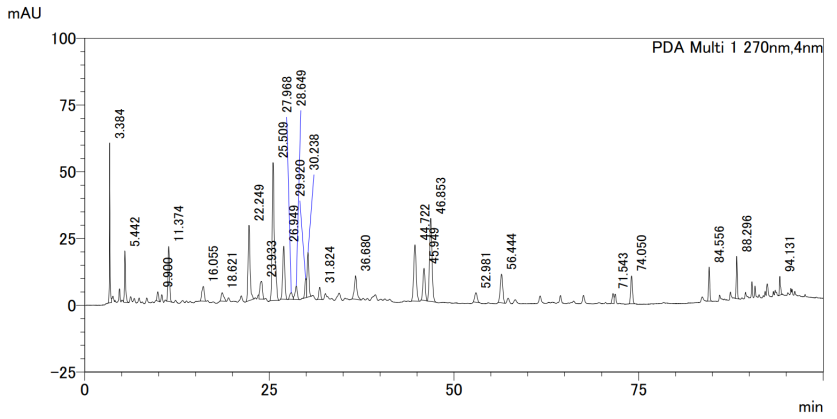


**Fig. S1** The chromatogram of the DH methanol extract (DHM)


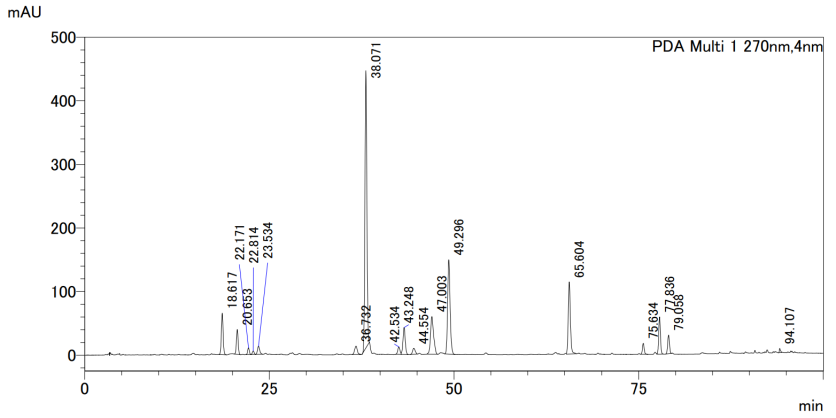


**Fig. S2** The chromatogram of the HQ methanol extract (HQM)


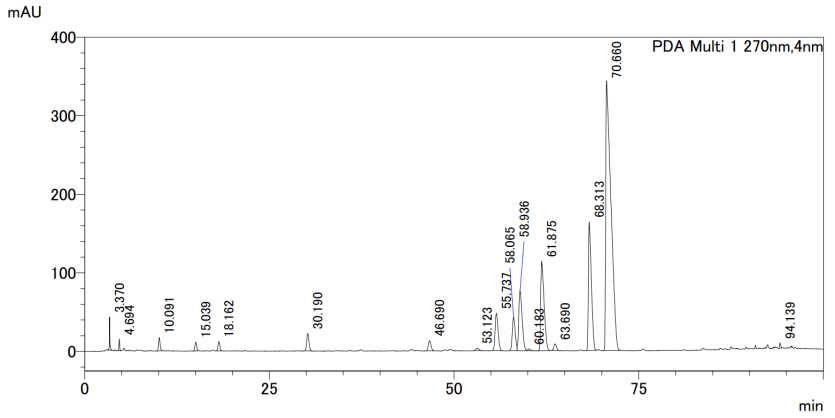


**Fig. S3** The chromatogram of the CHL methanol extract (CHLM)


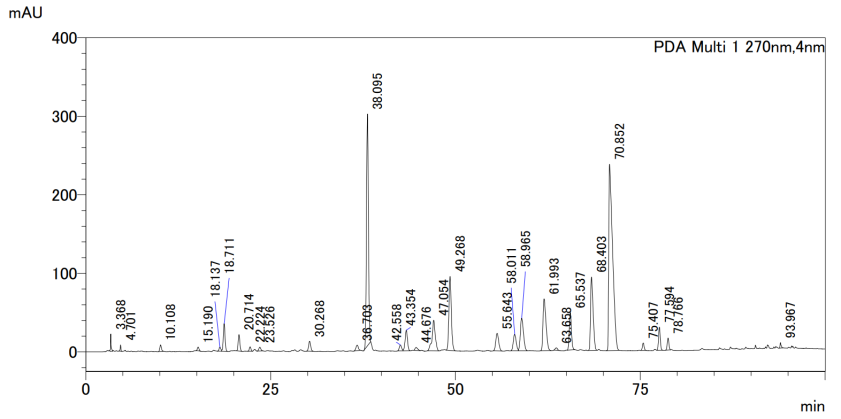


**Fig. S4** The chromatogram of the HQ and CHL methanol extract (HQHLM)


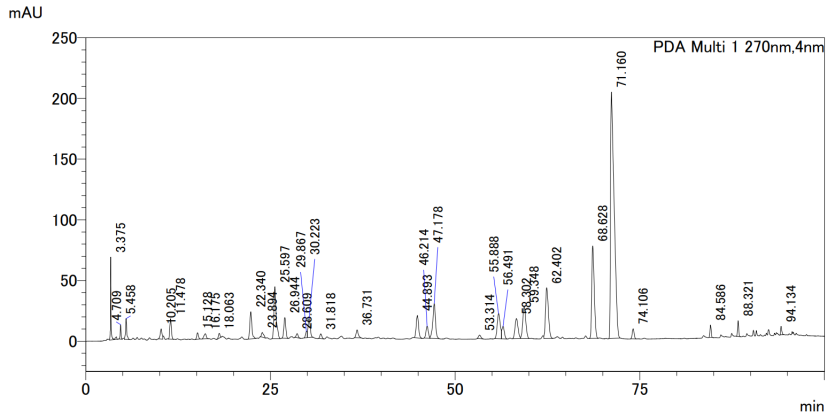


**Fig. S5** The chromatogram of the DH and CHL methanol extract (DHHLM)


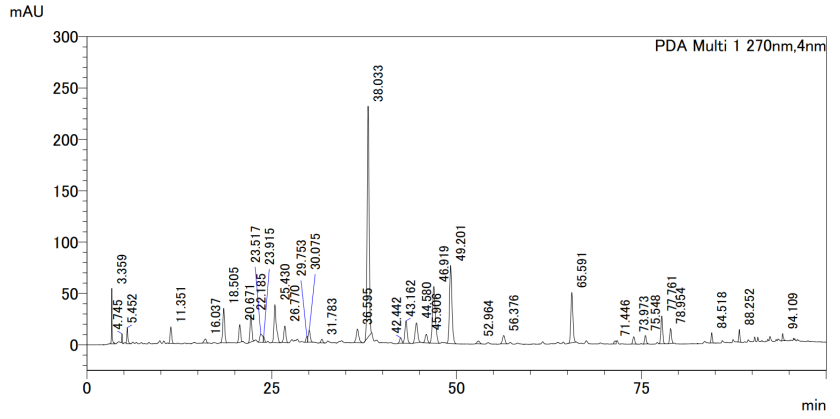


**Fig. S6** The chromatogram of the DH and HQ methanol extract (DHHQM)


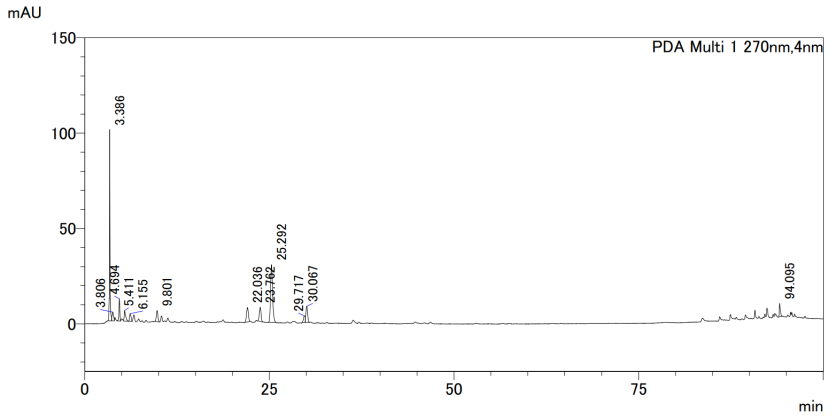


**Fig. S7** The chromatogram of the DHM water fraction (DHM-W)


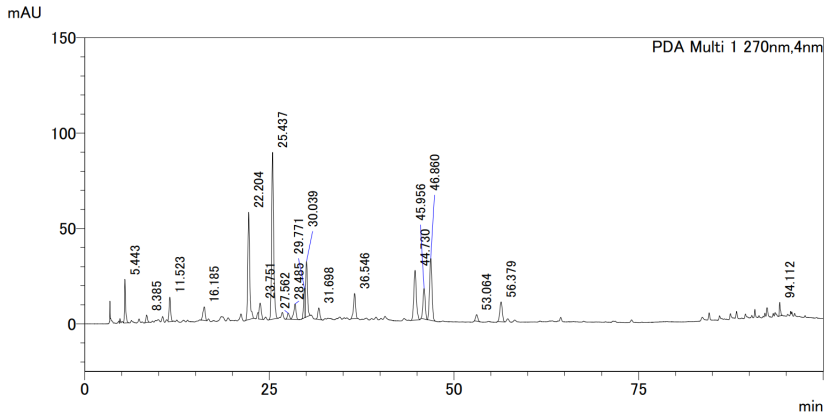


**Fig. S8** The chromatogram of the DHM n-butanol fraction (DHM-Bu)


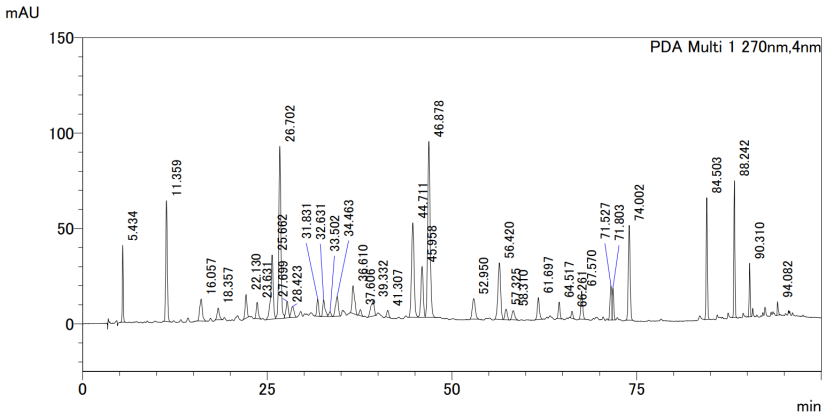


**Fig. S9** The chromatogram of the DHM ethyl acetate fraction (DHM-EA)


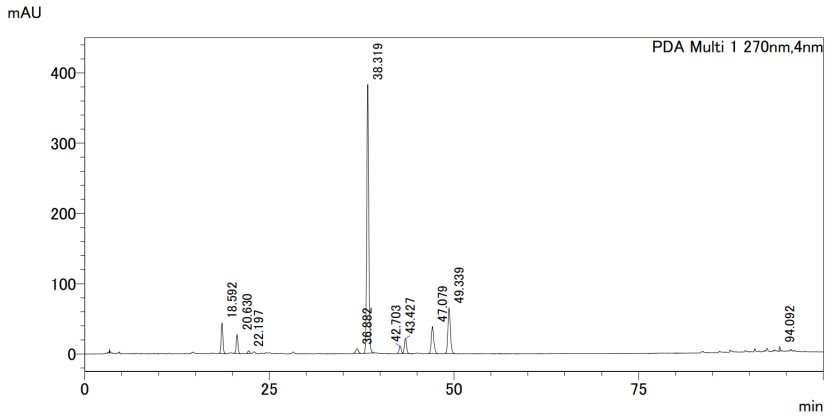


**Fig. S10** The chromatogram of the HQM water fraction (HQM-W)


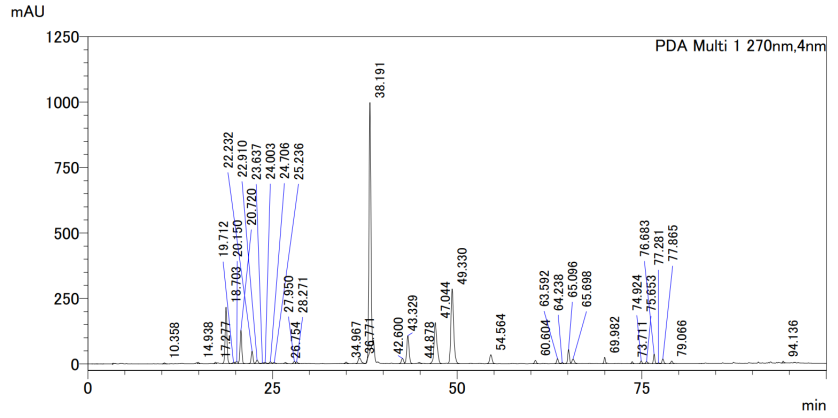


**Fig. S11** The chromatogram of the HQM n-butanol fraction (HQM-Bu)


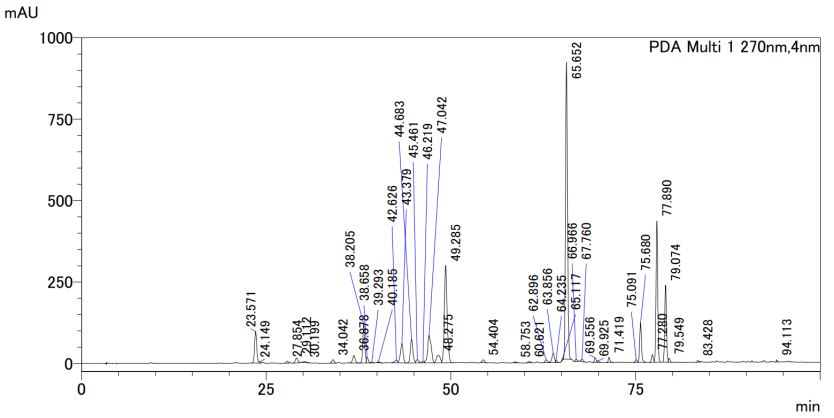


**Fig. S12** The chromatogram of the HQM ethyl acetate fraction (HQM-EA)


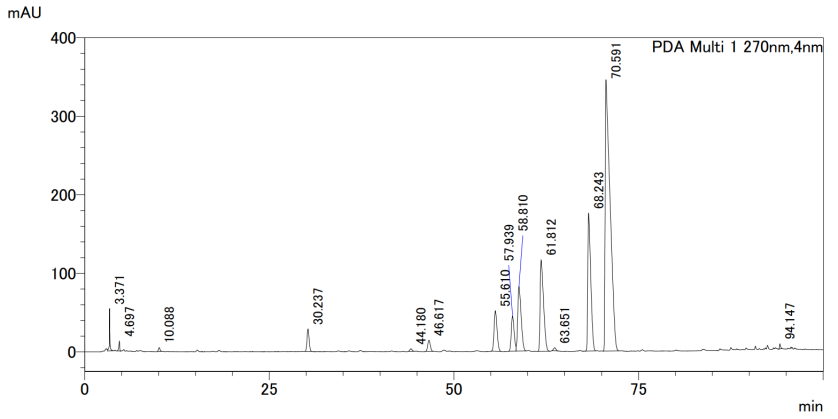


**Fig. S13** The chromatogram of the CHLM water fraction (CHLM-W)


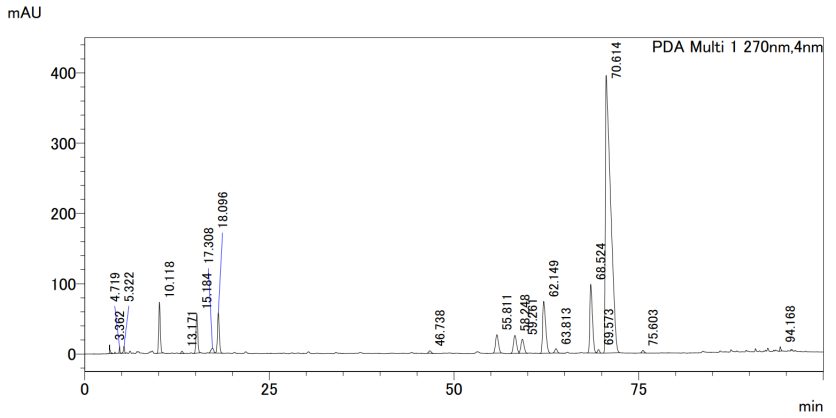


**Fig. S14** The chromatogram of the CHLM n-butanol fraction (CHLM-Bu)


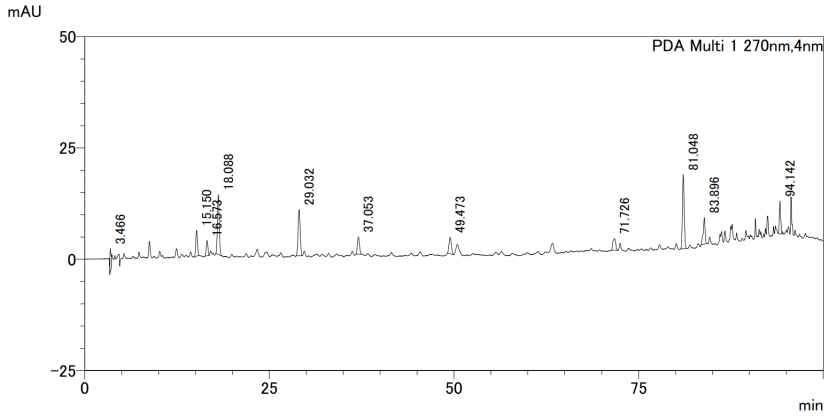


**Fig. S15** The chromatogram of the CHLM ethyl acetate fraction (CHLM-EA)


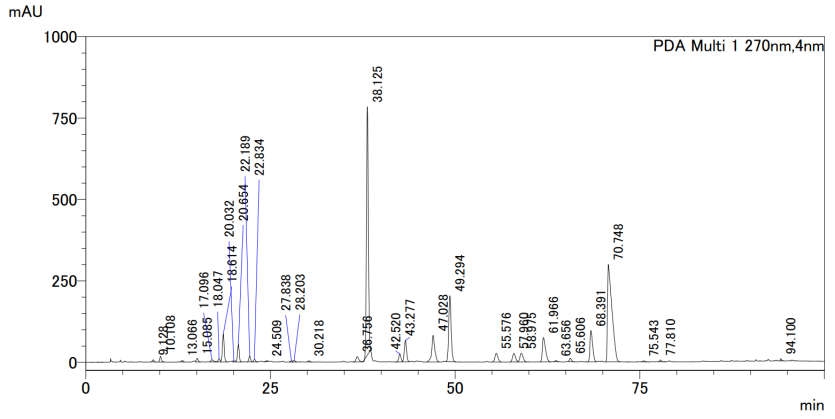


**Fig. S16** The chromatogram of the HQHLM water fraction (HQHLM-W)


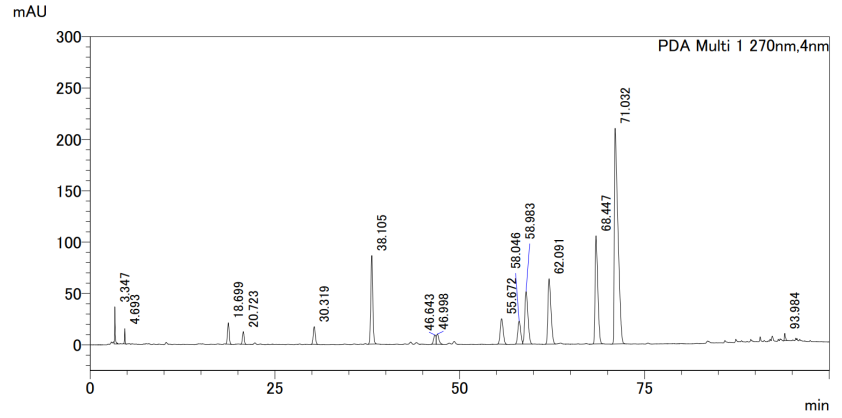


**Fig. S17** The chromatogram of the HQHLM n-butanol fraction (HQHLM-Bu)


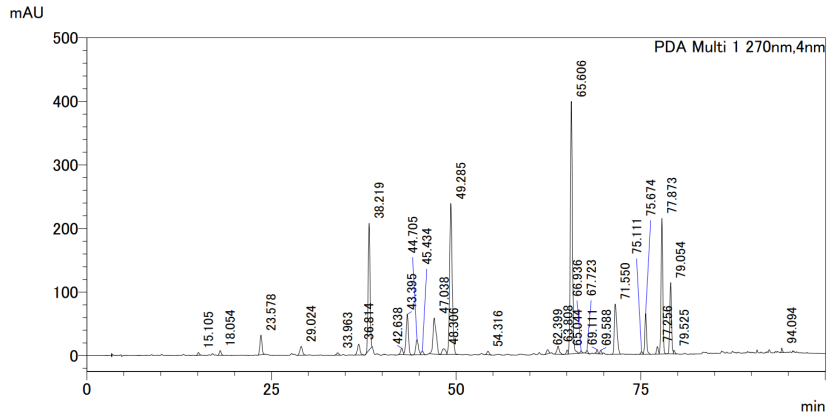


**Fig. S18** The chromatogram of the HQHLM ethyl acetate fraction (HQHLM-EA)


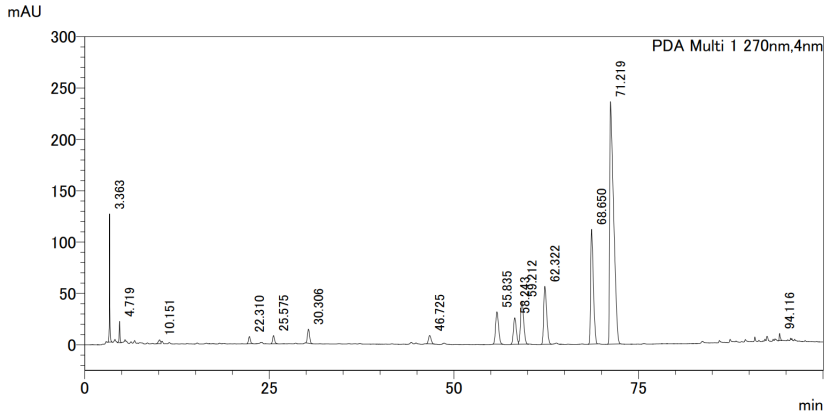


**Fig. S19** The chromatogram of the DHHLM water fraction (DHHLM-W)


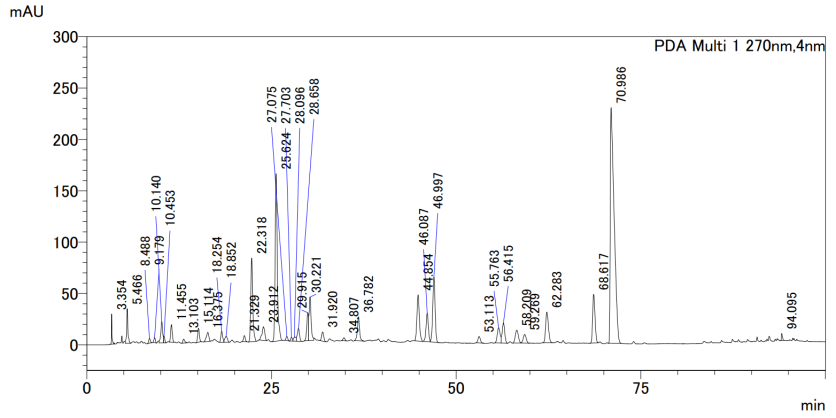


**Fig. S20** The chromatogram of the DHHLM n-butanol fraction (DHHLM-Bu)


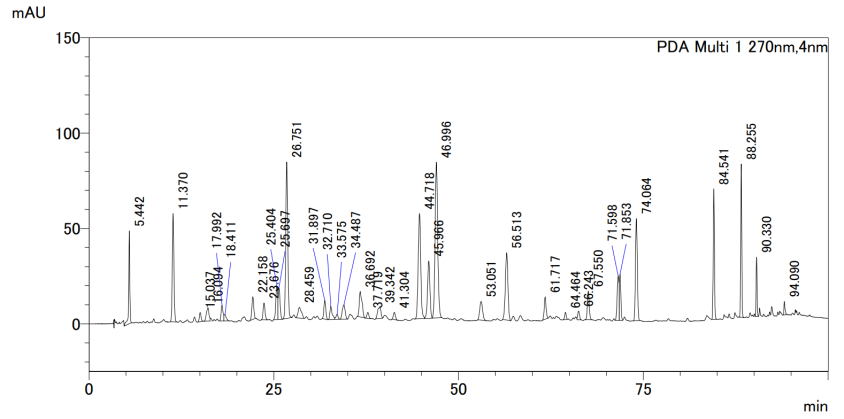


**Fig. S21** The chromatogram of the DHHLM ethyl acetate fraction (DHHLM-EA)


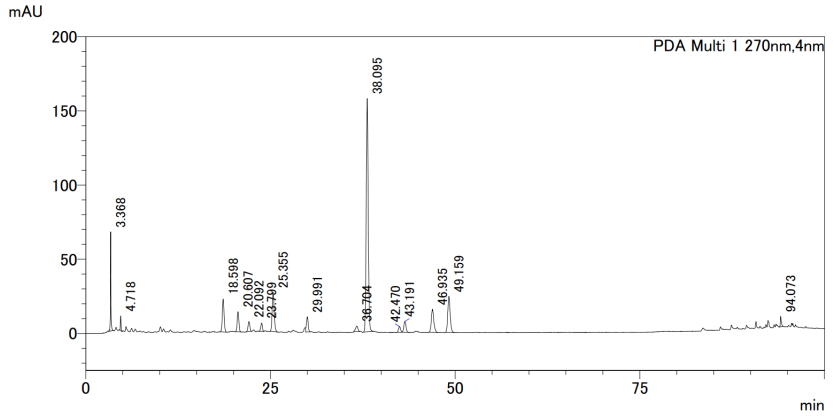


**Fig. S22** The chromatogram of the DHHQM water fraction (DHHQM-W)


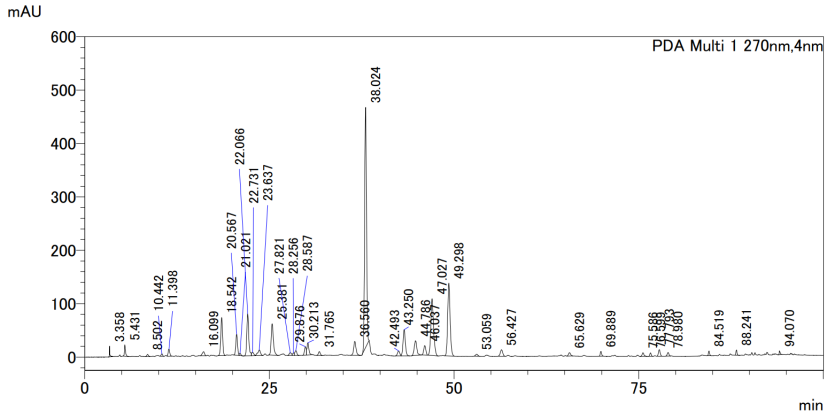


**Fig. S23** The chromatogram of the DHHQM n-butanol fraction (DHHQM-Bu)


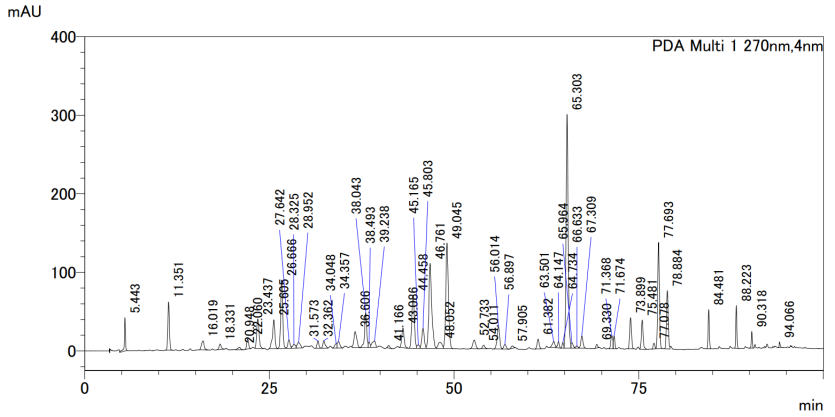


**Fig. S24** The chromatogram of the DHHQM ethyl acetate fraction (DHHQM-EA)

|  |
| --- |

**2. Effects of all samples on the High K^+^ and NA-induced vascular contractions**

**Table S2** The relaxant effects of all samples in NA and KCl-induced contractions in the vascular without endothelium

| Sample |  | KCl (relaxation %) | | | | |  | NA (relaxation %) | | | | | EC_50_ μg/mL | |
| --- | --- | --- | --- | --- | --- | --- | --- | --- | --- | --- | --- | --- | --- | --- |
|  | Con. | 1 μg | 3 μg | 10 μg | 30 μg | 100 μg |  | 1 μg | 3 μg | 10 μg | 30 μg | 100 μg | KCl | NA |
| CHL | M | 2.9 | 7.9 | 18.4 | 28.9 | 56.8 |  | 4.5 | 16.8 | 40.5 | 94.0 | 100.0 | 77.6 | 10.8 |
|  | W | 1.0 | 7.8 | 16.6 | 27.8 | 56.4 |  | 4.2 | 21.5 | 53.8 | 92.1 | 100.0 | 79.3 | 8.3 |
|  | Bu | 2.2 | 11.1 | 17.4 | 27.9 | 56.2 |  | 2.6 | 11.3 | 42.0 | 93.9 | 100.0 | 82.3 | 11.0 |
|  | EA | 2.3 | 8.1 | 13.7 | 28.4 | 55.2 |  | 0.4 | 4.0 | 10.6 | 15.7 | 50.9 | 83.3 | 104.2 |
| SHXXT | M | 6.4 | 11.3 | 17.5 | 31.0 | 62.7 |  | 0.6 | 7.1 | 21.2 | 86.3 | 100.0 | 65.1 | 16.2 |
|  | W | 4.5 | 9.1 | 13.6 | 20.3 | 30.8 |  | -1.0 | 5.6 | 29.0 | 61.7 | 97.5 | / | 20.0 |
|  | Bu | 4.88 | 10.5 | 16.2 | 26.6 | 45.1 |  | -1.5 | 10.5 | 73.7 | 90.9 | 100.0 | / | 6.7 |
|  | EA | 11.7 | 18.3 | 28.4 | 42.6 | 76.2 |  | 1.5 | 9.6 | 20.7 | 48.4 | 81.2 | 34.1 | 32.2 |
| HQ | M | 6.5 | 12.3 | 14.2 | 15.1 | 25.6 |  | 0.2 | 3.1 | 31.3 | 100.0 | 100.0 | / | 15.8 |
|  | W | 3.6 | 11.1 | 12.9 | 16.0 | 17.8 |  | 0.8 | 3.5 | 36.3 | 100.0 | 100.0 | / | 15.3 |
|  | Bu | 1.3 | 0.2 | 0.7 | 0.0 | 3.1 |  | 0.9 | 8.4 | 95.0 | 100.0 | 100.0 | / | 5.2 |
|  | EA | 2.6 | 6.6 | 12.4 | 26.1 | 74.4 |  | -3.2 | -0.2 | 10.9 | 65.2 | 100.0 | 57.5 | 23.3 |
| DH | M | 0.5 | 2.3 | 5.4 | 17.4 | 30.6 |  | 0.00 | 3.5 | 11.0 | 22.7 | 49.5 | / | / |
|  | W | 1.3 | 4.7 | 5.6 | 8.4 | 8.7 |  | 3.0 | 8.1 | 10.2 | 14.6 | 28.8 | / | / |
|  | Bu | 0.5 | 2.4 | 3.9 | 7.8 | 14.6 |  | 2.9 | 5.4 | 13.5 | 21.4 | 41.7 | / | / |
|  | EA | -0.4 | 2.8 | 6.8 | 18.2 | 47.7 |  | 1.6 | 11.6 | 34.6 | 90.0 | 100.0 | / | 12.8 |
| HQHL | M | 4.4 | 12.8 | 18.0 | 29.8 | 55.6 |  | 0.1 | 11.2 | 72.3 | 100.0 | 100.0 | 84.1 | 6.9 |
|  | W | 4.4 | 7.0 | 13.1 | 19.9 | 35.6 |  | 3.8 | 38.5 | 98.6 | 100.0 | 100.0 | / | 5.0 |
|  | Bu | 2.2 | 5.9 | 13.9 | 25.9 | 34.2 |  | -2.2 | 2.1 | 21.0 | 87.0 | 100.0 | / | 15.9 |
|  | EA | 3.7 | 5.7 | 10.7 | 24.3 | 72.4 |  | -4.2 | -3.7 | 10.9 | 93.3 | 100.0 | 61.1 | 15.9 |
| DHHL | M | 2.9 | 4.9 | 12.2 | 20.6 | 54.7 |  | 1.5 | 16.4 | 34.4 | 79.2 | 100.0 | 91.0 | 13.9 |
|  | W | 0.8 | 4.2 | 7.8 | 13.0 | 22.2 |  | 7.1 | 16.7 | 37.7 | 69.2 | 96.1 | / | 15.5 |
|  | Bu | 2.6 | 4.1 | 9.3 | 16.6 | 29.9 |  | 6.6 | 18.5 | 36.3 | 75.8 | 100.0 | / | 14.1 |
|  | EA | 3.5 | 5.5 | 8.6 | 28.6 | 67.0 |  | 1.8 | 8.4 | 30.4 | 70.6 | 100.0 | 60.8 | 17.4 |
| DHHQ | M | 0.7 | 1.4 | 9.9 | 10.4 | 16.4 |  | 7.7 | 4.8 | 9.6 | 44.0 | 90.7 | / | 33.8 |
|  | W | 1.0 | 2.2 | 3.6 | 5.3 | 4.6 |  | 7.7 | 9.8 | 13.0 | 62.3 | 95.0 | / | 24.5 |
|  | Bu | -0.6 | 1.4 | 7.2 | 11.0 | 20.7 |  | 12.5 | 12.0 | 26.8 | 76.5 | 98.3 | / | 17.0 |
|  | EA | 0.0 | 2.8 | 4.2 | 9.0 | 24.7 |  | 3.4 | 10.9 | 17.1 | 53.8 | 93.8 | / | 28.8 |

Each value represents the mean of 4–6 experiments.

**3. Sanoshashinto water extraction data**

A blended mixture of DH, HQ, and CHL in 1:1:1 ratio was refluxed with water for 1.5 h, and this procedure was repeated three times. The product collected by refluxing was filtered. The filtrate was concentrated under reduced pressure at 60 ℃ to obtain the solid extract (yield: 32.7%).


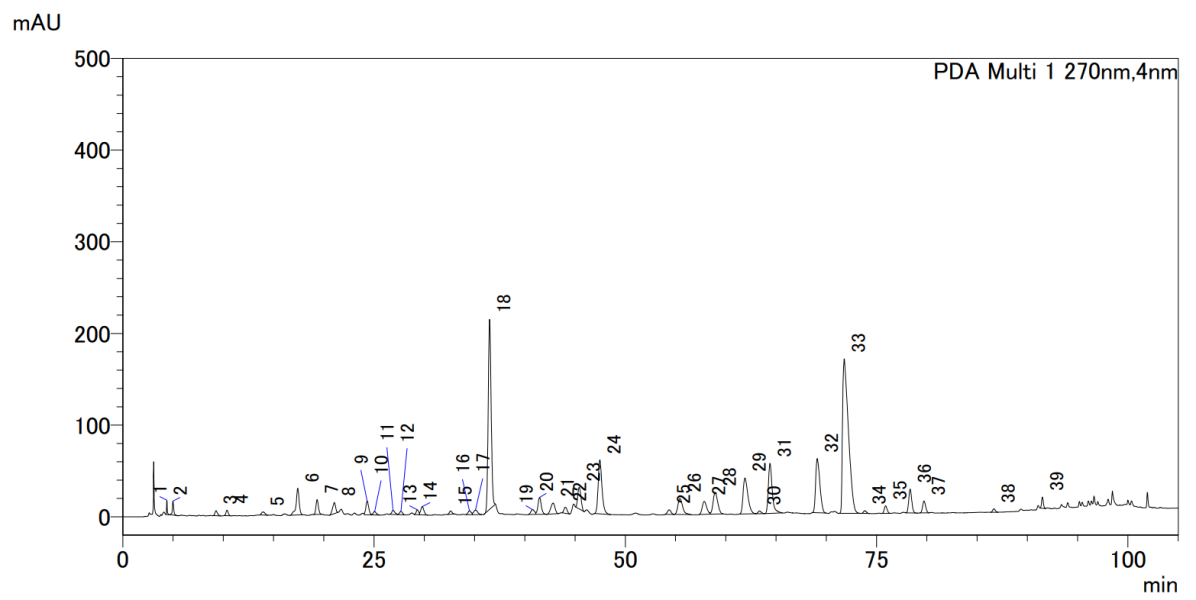


**Fig. S25** The chromatogram of the SHXXTM


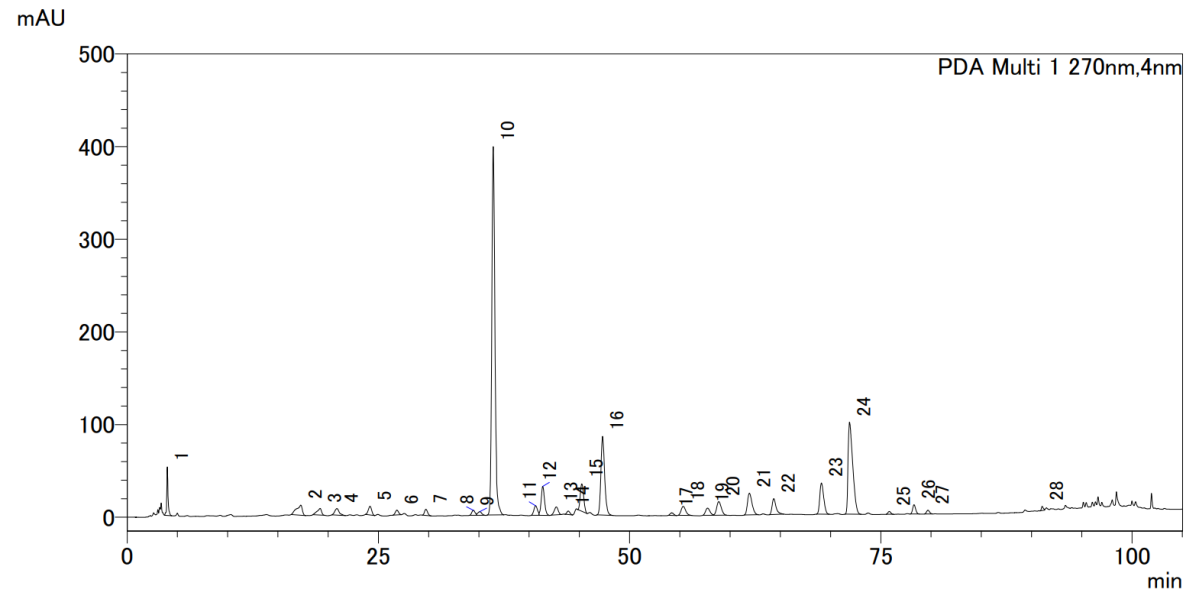


**Fig. S26** The chromatogram of the SHXXTW

**Table S3** The peak corresponding relation between SHXXTM and SHXXTW

| Peak No and name  Sample name | SHXXTM | SHXXT W |
| --- | --- | --- |
|  | 1 |  |
|  | 2 | 1 |
|  | 3 |  |
|  | 4 |  |
|  | 5  2.9  7.9  18.4  28.9  56.8 | 16.8  40.5  94.0  100.0  77.6  10.8 |
|  | 6 | 2 |
|  | 7 | 3 |
|  | 8 | 4 |
|  | 9 | 5 |
|  | 10 |  |
|  | 11 | 6 |
|  | 12 |  |
|  | 13 |  |
| sennoside A | 14 | 7 |
|  | 15 |  |
|  | 16 | 8 |
|  | 17 | 9 |
| baicalin | 18 | 10 |
|  | 19 | 11 |
|  | 20 | 12 |
|  | 21 | 13 |
|  | 22 | 14 |
|  | 23 | 15 |
| wogonoside | 24 | 16 |
|  | 25 | 17 |
|  | 26 | 18 |
|  | 27 | 19 |
|  | 28 | 20 |
| coptisine | 29 | 21 |
|  | 30 |  |
| baicalein | 31 | 22 |
| palmatine | 32 | 23 |
| berberine | 33 | 24 |
| rhein | 34 | 25 |
|  | 35 |  |
|  | 36 | 26 |
| wogonin | 37 | 27 |
| emodin | 38 |  |
| chrysophanic acid | 39 | 28 |

**Table S4** The EC_50_ values of SHXXTW and SHXXTM

| **Extracts name** | EC_50_ μg/mL (NA-induced contraction) |
| --- | --- |
| SHXXTW | 11.6 |
| SHXXTM | 16.2 |


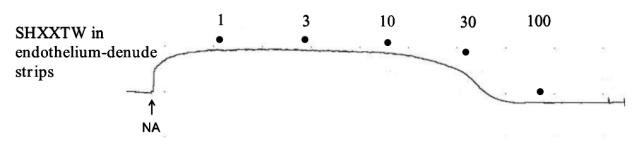


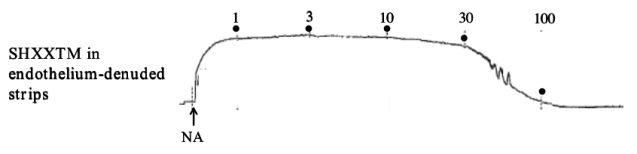


Fig. S27 The SHXXTW and SHXXTM in NA-induced contractions of endothelium-denuded strips.

1. **Principal component regression (PCR) results**

**a**


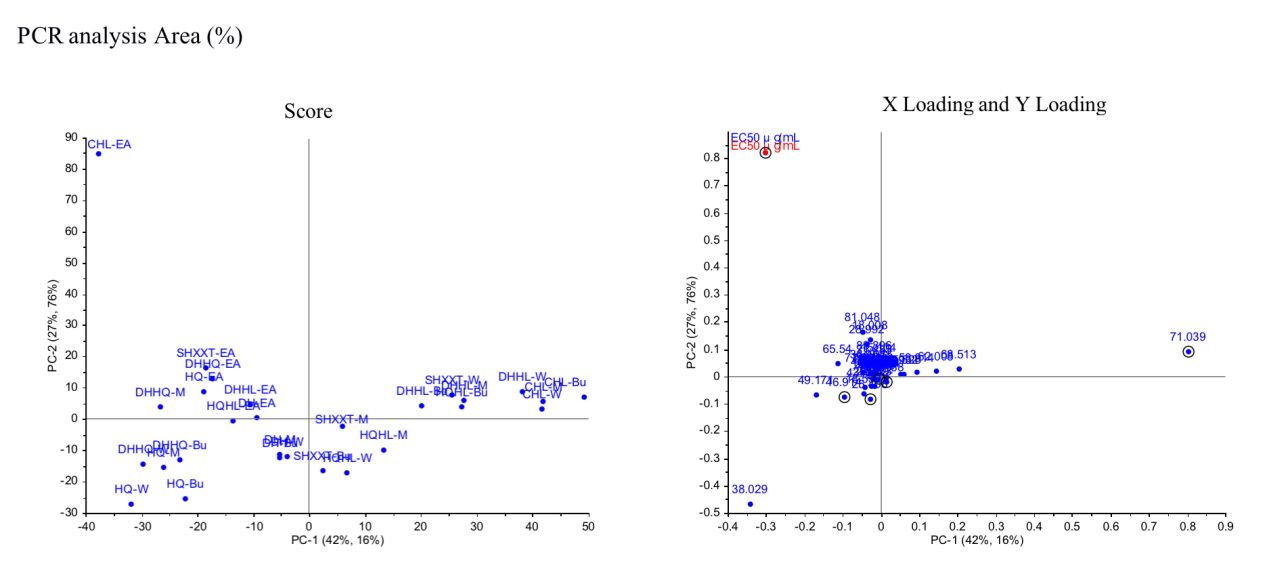


**b**


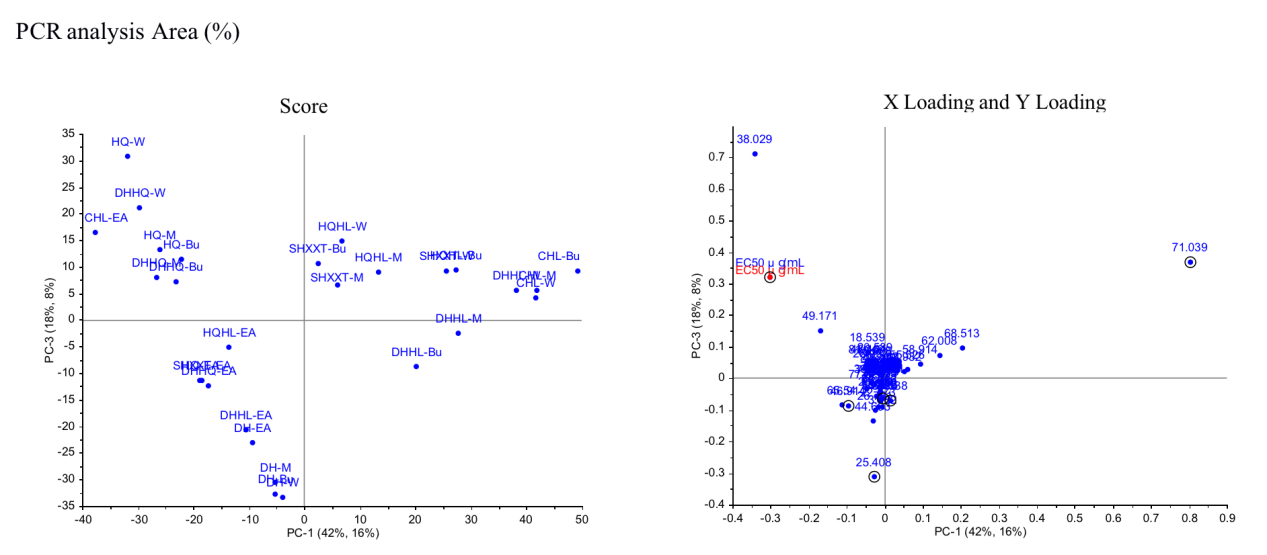


**c**


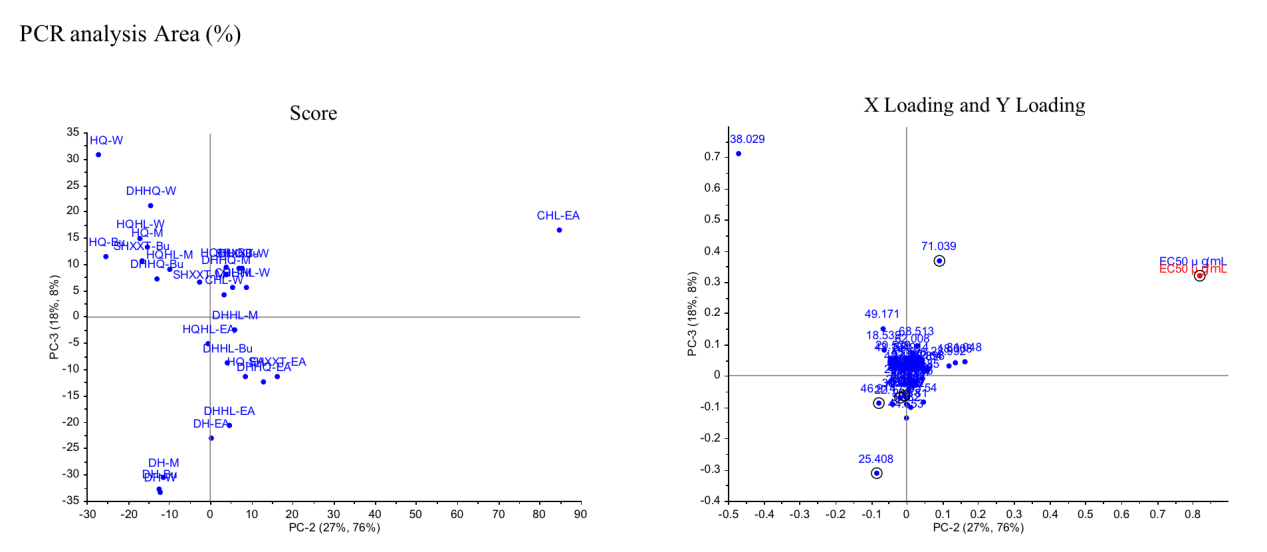


**d**


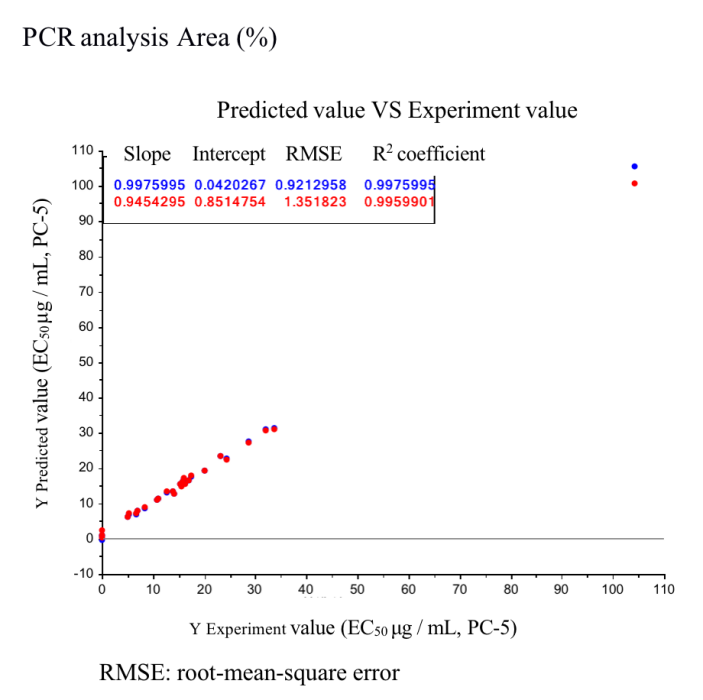


Fig. S28 Score and Loading plots of disassembled SHXXT samples and EC_50_ data by PCR

a) Score and Loading plots of disassembled SHXXT samples and EC_50_ data in PC-1 and PC-2

b) Score and Loading plots of disassembled SHXXT samples and EC_50_ data in PC-1 and PC-3

c) Score and Loading plots of disassembled SHXXT samples and EC_50_ data in PC-2 and PC-3

d) PCR results
