# Supplementary material for: Antihypertensive constituents in Sanoshashinto
Source: J Nat Med. 2020 Jan 1;74(2):421–33. doi: 10.1007/s11418-019-01382-9 (PMC7929964; doi:10.1007/s11418-019-01382-9)

Sample	Row	1	2	3	4	5	6	7	8	9	10	11	12	13	14	15	16	17	18	19	20
																			Blin		
	Peak No.	1	2	3	4	5	6	7	8	9	10	11	12	13	14	15	16	17	18	19	20
SHXXT-M	RT (retention time)	3.382	4.741	5.442	10.21	11.482	15.104	16.075	18.008	18.539	20.539	22.173	23.501	25.408	26.781	29.813	30.138	36.464	38.029	42.427	43.164
	RRT (relative retention time)	0.0359	0.0504	0.0578	0.1085	0.1220	0.1605	0.1708	0.1914	0.1970	0.2183	0.2356	0.2498	0.2700	0.2846	0.3168	0.3203	0.3875	0.4042	0.4509	0.4587
	Area	230594	62813	115101	100303	185139	53368	69207	54438	436058	209226	338248	199840	651677	233221	64746	296855	241075	3863931	90547	368406
	Area%	1.041	0.284	0.52	0.453	0.836	0.241	0.312	0.246	1.968	0.944	1.527	0.902	2.941	1.053	0.292	1.34	1.088	17.44	0.409	1.663
	Peak No.	1	2	3	4	5	6	7	8	9	10	11	12	13	14	15	16	17	18	19	20
SHXXTM-W	RT	3.372	4.74							18.634	20.611	22.075		25.388			30.056		37.816		
	RRT	0.0358	0.0504							0.1981	0.2191	0.2346		0.2698			0.3195		0.4019		
	Area	458349	126041							304584	174177	84393		151533			218644		991774		
	Area%	3.741	1.029							2.486	1.422	0.689		1.237			1.785		8.096		
	Peak No.	1	2	3	4	5	6	7	8	9	10	11	12	13	14	15	16	17	18	19	20
SHXXTMBu	RT	3.369	5.429		10.223	11.449	15.12	16.175	18.061	18.62	20.637	22.129		25.598		29.893	30.225	36.59	38.102	42.594	43.329
	RRT	0.0358	0.0577		0.1087	0.1217	0.1607	0.1719	0.1920	0.1979	0.2194	0.2352		0.2721		0.3177	0.3213	0.3889	0.4050	0.4528	0.4606
	Area	83957	140113		206954	98755	87477	93904	68337	734974	400007	802717		130282		195089	429150	359892	8281831	206722	738291
	Area%	0.293	0.489		0.722	0.345	0.305	0.328	0.239	2.566	1.396	2.802		4.548		0.681	1.498	1.256	28.911	0.722	2.577
	Peak No.	1	2	3	4	5	6	7	8	9	10	11	12	13	14	15	16	17	18	19	20
SHXXTMEA	RT		5.44			11.358	15.052	16.108	18.004			22.313	23.563	25.562	26.862			36.636	38.106	42.547	43.293
	RRT		0.0578			0.1208	0.1600	0.1713	0.1914			0.2372	0.2505	0.2718	0.2856			0.3895	0.4052	0.4524	0.4603
	Area		400759			599068	78876	206869	92765			331526	667462	294455	1062047			493241	614428	52441	348462
	Area%		1.481			2.214	0.291	0.764	0.343			1.225	2.467	1.088	3.925			1.823	2.271	0.194	1.288
	Peak No.	1	2	3	4	5	6	7	8	9	10	11	12	13	14	15	16	17	18	19	20
CHLM	RT	3.37	4.694		10.091		15.039		18.162								30.19				
	RRT	0.0358	0.0499		0.1072		0.1598		0.1929								0.3207				
	Area	188336	100414		244226		180245		207009								481027				
	Area%	0.592	0.316		0.768		0.567		0.651								1.512				
	Peak No.	1	2	3	4	5	6	7	8	9	10	11	12	13	14	15	16	17	18	19	20
CHLMW	RT	3.371	4.697		10.088												30.237				
	RRT	0.0358	0.0499		0.1072												0.3212				
	Area	268973	103881		71835												561246				
	Area%	0.853	0.33		0.228												1.781				
	Peak No.	1	2	3	4	5	6	7	8	9	10	11	12	13	14	15	16	17	18	19	20
CHLM-Bu	RT	3.362	4.719	5.322	10.118		15.184		18.096												
	RRT	0.0357	0.0501	0.0565	0.1074		0.1612		0.1922												
	Area	74772	55075	100197	1057469		890278		100117												
	Area%	0.252	0.185	0.337	3.558		2.995		3.368												
	Peak No.	1	2	3	4	5	6	7	8	9	10	11	12	13	14	15	16	17	18	19	20
CHLM-EA	RT	3.466					15.15		18.088												
	RRT	0.0368					0.1609		0.1921												
	Area	52613					89376		232521												
	Area%	3.945					6.701		17.433												
	Peak No.	1	2	3	4	5	6	7	8	9	10	11	12	13	14	15	16	17	18	19	20
HQM	RT									18.617	20.653	22.171	23.534					36.732	38.071	42.534	43.248
	RRT									0.1978	0.2195	0.2356	0.2501					0.3903	0.4046	0.4520	0.4596
	Area									1082084	646660	164938	264716					333670	7919887	240557	907790
	Area%									5.124	3.015	0.781	1.254					1.58	37.505	1.139	4.299
	Peak No.	1	2	3	4	5	6	7	8	9	10	11	12	13	14	15	16	17	18	19	20
HQM-W	RT									18.592	20.63	22.197						36.882	38.319	42.703	43.427
	RRT									0.1976	0.2193	0.2359						0.3920	0.4073	0.4538	0.4615
	Area									709500	440469	65630						171105	7136885	209635	437046
	Area%									6.087	3.779	0.563						1.468	61.226	1.798	3.749
	Peak No.	1	2	3	4	5	6	7	8	9	10	11	12	13	14	15	16	17	18	19	20
HQMBu	RT				10.358		14.938			18.703	20.72	22.232	23.637	25.236	26.754			36.771	38.191	42.6	43.329
	RRT				0.1100		0.1587			0.1987	0.2201	0.2362	0.2511	0.2681	0.2842			0.3906	0.4057	0.4525	0.4603
	Area				56886		73517			3571275	212594	778637	52698	66303	58991			602353	16760086	419803	228101
	Area%				0.129		0.167			8.125	4.836	1.771	0.12	0.151	0.134			1.37	38.129	0.955	5.189
	Peak No.	1	2	3	4	5	6	7	8	9	10	11	12	13	14	15	16	17	18	19	20
HQMEA	RT												23.571				30.199	36.878	38.205	42.626	43.379
	RRT												0.2505				0.3209	0.3918	0.4059	0.4529	0.4609
	Area												197343				93044	548271	2230821	222808	153544
	Area%												3.596				0.17	0.999	4.065	0.406	2.798
	Peak No.	1	2	3	4	5	6	7	8	9	10	11	12	13	14	15	16	17	18	19	20
DHM	RT	3.384		5.442		11.374		16.055		18.621		22.249	23.933	25.509	26.949	29.92	30.238	36.68			
	RRT	0.0359		0.0578		0.1208		0.1706		0.1978		0.2364	0.2543	0.2710	0.2863	0.3179	0.3212	0.3897			
	Area	265623		196287		298432		124724		76740		76740	160758	115266	393115	104478	275424	210121			
	Area%	4.088		3.021		4.593		4.593		1.181		1.181	2.474	17.739	6.05	1.608	4.239	3.234			
	Peak No.	1	2	3	4	5	6	7	8	9	10	11		13	14	15	16	17	18	19	20
DHM-W	RT	3.386	4.694	5.411								22.036	23.762	25.292		29.717	30.067				
	RRT	0.0360	0.0499	0.0575								0.2342	0.2525	0.2688		0.3158	0.3195				
	Area	459801	95599	76373								133874	120104	574633		58110	135332				
	Area%	23.889	4.967	3.968								6.955	6.24	29.855		3.019	7.031				
	Peak No.	1	2	3	4	5	6	7	8	9	10	11		13	14	15	16	17	18	19	20
DHMBu	RT			5.443		11.523		16.185				22.204	23.751	25.437		29.771	30.039	36.546			
	RRT			0.0578		0.1224		0.1720				0.2359	0.2524	0.2703		0.3163	0.3192	0.3883			
	Area			237012		188096		147782				101631	164720	165511		211521	509386	257344			
	Area%			3.452		2.74		2.153				14.804	2.399	24.109		3.081	7.42	3.749			
	Peak No.	1	2	3	4	5	6	7	8	9	10	11		13	14	15	16	17	18	19	20
DHMEA	RT			5.434		11.359		16.057		18.357		22.13	23.631	25.662	26.702			36.61			
	RRT			0.0578		0.1207		0.1707		0.1951		0.2352	0.2512	0.2728	0.2838			0.3891			
	Area			349931		928600		286804		127687		219619	160137	775817	1771926			346364			
	Area%			2.284		6.06		1.872		0.833		1.433	1.045	5.063	11.564			2.261			
	Peak No.	1	2	3	4	5	6	7	8	9	10	11	12	13	14	15	16	17	18	19	20
HQHLM	RT	3.368	4.701		10.108		15.19		18.137	18.711	20.714	22.224	23.526				30.268	36.703	38.095	42.558	43.354
	RRT	0.0358	0.0500		0.1076		0.1617		0.1930	0.1991	0.2204	0.2365	0.2504				0.3221	0.3906	0.4054	0.4529	0.4614
	Area	108689	52182		125871		74637		88067	578648	337184	84422	92917				244335	155471	5397723	161797	589047
	Area%	0.382	0.184		0.443		0.263		0.31	2.035	1.186	0.297	0.327				0.859	0.547	18.984	0.569	2.072
	Peak No.	1	2	3	4	5	6	7	8	9	10	11	12	13	14	15	16	17	18	19	20
HQHLMW	RT				10.108		15.085		18.047	18.614	20.654	22.189					30.218	36.756	38.125	42.52	43.277
	RRT				0.1074		0.1603		0.1918	0.1978	0.2195	0.2358					0.3211	0.3906	0.4052	0.4519	0.4599
	Area				278376		184596		162973	1451430	884313	279584					60264	406472	13433326	503187	137447
	Area%				0.601		0.398		0.352	3.133	1.909	0.604					0.13	0.877	28.998	1.086	2.967
	Peak No.	1	2	3	4	5	6	7	8	9	10	11	12	13	14	15	16	17	18	19	20
HQHLM-Bu	RT	3.347	4.693							18.699	20.723						30.319		38.105		
	RRT	0.0356	0.0499							0.1990	0.2205						0.3226		0.4054		
	Area	162746	77369							333430	197369						332410		1610500		
	Area%	0.927	0.44							1.898	1.124						1.892		9.168		
	Peak No.	1	2	3	4	5	6	7	8	9	10	11	12	13	14	15	16	17	18	19	20
HQHLM-EA	RT						15.105		18.054				23.578					36.814	38.219	42.638	43.395
	RRT						0.1605		0.1919				0.2506					0.3912	0.4062	0.4531	0.4612
	Area						68315		127357				598177					409114	3568327	205444	139591
	Area%						0.209		0.39				1.831					1.252	10.922	0.629	4.273
DHHQM	Peak No.	1	2	3	4	5	6	7	8	9	10	11	12	13	14	15	16	17	18	19	20
	RT	3.359	4.745	5.452		11.351		16.037		18.505	20.671	22.185	23.517	25.43	26.77	29.753	30.075	36.595	38.033	42.442	43.162
	RRT	0.0357	0.0504	0.0579		0.1206		0.1704		0.1966	0.2196	0.2357	0.2499	0.2702	0.2845	0.3162	0.3196	0.3889	0.4041	0.4510	0.4586
	Area	237077	51031	155277		237129		87673		588447	285574	448835	214325	804951	303350	79659	218359	319110	4151525	103266	465174
	Area%	1.557	0.335	1.019		1.557		0.576		3.863	1.875	2.947	1.407	5.285	1.992	0.523	1.434	2.095	27.257	0.678	3.054
DHHQM-W	Peak No.	1	2	3	4	5	6	7	8	9	10	11	12	13	14	15	16	17	18	19	20
	RT	3.368	4.718							18.598	20.607	22.092	23.799	25.355		29.991		36.704	38.095	42.47	43.191
	RRT	0.0358	0.0502							0.1977	0.2191	0.2348	0.2530	0.2695		0.3188		0.3902	0.4050	0.4515	0.4591
	Area	280401	57812							368372	215629	116039	87058	499986		166966		86450	2927707	72481	145316
	Area%	4.672	0.963							6.138	3.593	1.933	1.451	8.331		2.782		1.44	48.78	1.208	2.421
DHHQM-Bu	Peak No.	1	2	3	4	5	6	7	8	9	10	11	12	13	14	15	16	17	18	19	20
	RT	3.358		5.431		11.398		16.099		18.542	20.567	22.066	23.637	25.381		29.876	30.213	36.56	38.024	42.493	43.25
	RRT	0.0357		0.0577		0.1212		0.1711		0.1971	0.2186	0.2346	0.2513	0.2698		0.3176	0.3212	0.3886	0.4042	0.4517	0.4598
	Area	89226		215038		200289		161038		1224855	640531	146064	272700	121721		254286	397142	590156	8068352	192236	105403
	Area%	0.356		0.857		0.799		0.642		4.884	2.554	5.824	1.087	4.853		1.014	1.584	2.353	32.171	0.767	4.203
DHHQM-EA	Peak No.	1	2	3	4	5	6	7	8	9	10	11	12	13	14	15	16	17	18	19	20
	RT			5.443		11.351		16.019		18.331		22.06	23.437	25.605	26.666			36.606	38.043		43.086
	RRT			0.0579		0.1207		0.1703		0.1949		0.2345	0.2492	0.2722	0.2835			0.3892	0.4044		0.4580
	Area			441910		911382		290122		133897		231842	834153	808759	1660419			552479	890504		569537
	Area%			1.34		2.763		0.88		0.406		0.703	2.529	2.452	5.035			1.675	2.7		1.727
DHHLM	Peak No.	1	2	3	4	5	6	7	8	9	10	11	12	13	14	15	16	17	18	19	20
	RT	3.375	4.709	5.458	10.205	11.478	15.128	16.175	18.063			22.34		25.597	26.944	29.867	30.223	36.731			
	RRT	0.0359	0.0500	0.0580	0.1084	0.1219	0.1607	0.1718	0.1919			0.2373		0.2719	0.2862	0.3173	0.3211	0.3902			
	Area	325681	88821	191771	137671	243726	83027	87051	58566			430732		906124	316968	77732	322034	130714			
	Area%	1.885	0.514	1.11	0.797	1.411	0.481	0.504	0.339			2.493		5.245	1.835	0.45	1.864	0.757			
	Peak No.	1	2	3	4	5	6	7	8	9	10	11	12	13	14	15	16	17	18	19	20

Sample	Row	21	22	23	24	25	26	27	28	29	30	31	32	33	34	35	36	37	38	39	40
												Blein	Pal	Ber							NotInput
	Peak No.	21	22	23	24	25	26	27	28	29	30	31	32	33	34	35	36	37	38	39	40
SHXXT-M	RT (retention time)	44.653	45.926	46.914	49.171	52.85	55.526	56.229	57.932	58.914	62.008	65.54	68.513	71.039	73.966	75.617	77.824	79.019	84.512	88.241	94.096
	RRT (relative retention time)	0.4745	0.4881	0.4986	0.5226	0.5617	0.5901	0.5976	0.6157	0.6261	0.6590	0.6965	0.7281	0.7550	0.7861	0.8036	0.8271	0.8398	0.8981	0.9378	
	Area	357505	143628	126423	1384335	51189	423109	151558	360092	654664	103061	680205	1355080	5415022	98901	95601	323929	172460	85715	108594	
	Area%	1.614	0.648	5.706	6.248	0.231	1.91	0.684	1.625	2.955	4.652	3.07	6.116	24.441	0.446	0.432	1.462	0.778	0.387	0.49	
	Peak No.	21	22	23	24	25	26	27	28	29	30	31	32	33	34	35	36	37	38	39	40
SHXXTM-W	RT				49.059		55.439		57.809	58.78	61.929		68.43	71.035							94.084
	RRT				0.5214		0.5893		0.6144	0.6248	0.6582		0.7273	0.7550							
	Area				79611		493847		393859	841698	109762		1586229	4938815							
	Area%				0.65		4.031		3.215	6.871	8.96		12.948	40.314							
	Peak No.	21	22	23	24	25	26	27	28	29	30	31	32	33	34	35	36	37	38	39	40
SHXXTMBu	RT	44.762	45.969	46.982	49.224		55.701	56.371	58.116	59.109	62.132	65.523	68.572	71.034							94.078
	RRT	0.4758	0.4886	0.4994	0.5232		0.5921	0.5992	0.6177	0.6283	0.6604	0.6965	0.7289	0.7551							
	Area	279693	124329	188612	1977930		363709	93625	348989	349837	842061	54793	1205141	6385395							
	Area%	0.976	0.434	6.584	6.905		1.27	0.327	1.218	1.221	2.94	0.191	4.207	22.291							
	Peak No.	21	22	23	24	25	26	27	28	29	30	31	32	33	34	35	36	37	38	39	40
SHXXTMEA	RT	44.8	46.082	47.039	49.372	53.042		56.476			61.716	65.563	68.421		74.015	75.6	77.815	79.002	84.51	88.222	94.051
	RRT	0.4763	0.4900	0.5001	0.5249	0.5640		0.6005			0.6562	0.6971	0.7275		0.7870	0.8038	0.8274	0.8400	0.8986	0.9380	
	Area	146311	535604	216762	2448285	174976		636684			177594	4424626	91612		503506	606641	189256	102989	686894	526174	
	Area%	5.407	1.979	8.01	9.047	0.647		2.353			0.656	16.351	0.339		1.861	2.242	6.994	3.806	2.538	1.944	
	Peak No.	21	22	23	24	25	26	27	28	29	30	31	32	33	34	35	36	37	38	39	40
CHLM	RT			46.69		53.123	55.737		58.065	58.936	61.875		68.313	70.66							94.139
	RRT			0.4960		0.5643	0.5921		0.6168	0.6261	0.6573		0.7257	0.7506							
	Area			322406		82637	131903		114924	2397953	359341		4529627	16706289							
	Area%			1.013		0.26	4.146		3.613	7.538	11.296		14.238	52.515							
	Peak No.	21	22	23	24	25	26	27	28	29	30	31	32	33	34	35	36	37	38	39	40
CHLMW	RT	44.18		46.617			55.61		57.939	58.81	61.812		68.243	70.591							94.147
	RRT	0.4693		0.4952			0.5907		0.6154	0.6247	0.6565		0.7249	0.7498							
	Area	69167		339603			144285		121125	2636113	371419		4912842	16037568							
	Area%	0.219		1.077			4.578		3.843	8.364	11.784		15.587	50.882							
	Peak No.	21	22	23	24	25	26	27	28	29	30	31	32	33	34	35	36	37	38	39	40
CHLM-Bu	RT			46.738			55.811		58.248	59.261	62.149		68.524	70.614		75.603					94.168
	RRT			0.4963			0.5927		0.6186	0.6293	0.6600		0.7277	0.7499		0.8029					
	Area			79666			720023		740412	576959	211645		2363654	19354611		74412					
	Area%			0.268			2.422		2.491	1.941	7.121		7.952	65.116		0.25					
	Peak No.	21	22	23	24	25	26	27	28	29	30	31	32	33	34	35	36	37	38	39	40
CHLM-EA	RT				49.473																94.142
	RRT				0.5255																
	Area				81153																
	Area%				6.084																
	Peak No.	21	22	23	24	25	26	27	28	29	30	31	32	33	34	35	36	37	38	39	40
HQM	RT	44.554		47.003	49.296							65.604				75.634	77.836	79.058			94.107
	RRT	0.4734		0.4995	0.5238							0.6971				0.8037	0.8271	0.8401			
	Area	229400		171062	3453591							2274898				286864	960893	495479			
	Area%	1.086		8.101	16.355							10.773				1.358	4.55	2.346			
	Peak No.	21	22	23	24	25	26	27	28	29	30	31	32	33	34	35	36	37	38	39	40
HQM-W	RT			47.079	49.339																94.092
	RRT			0.5004	0.5244																
	Area			905579	1515673																
	Area%			7.769	13.003																
	Peak No.	21	22	23	24	25	26	27	28	29	30	31	32	33	34	35	36	37	38	39	40
HQMBu	RT	44.878		47.044	49.33							65.698				75.653	77.865	79.066			94.136
	RRT	0.4767		0.4997	0.5240							0.6979				0.8037	0.8272	0.8399			
	Area	50827		426565	7285245							367258				134231	293553	152205			
	Area%	0.116		9.704	16.574							0.836				0.305	0.668	0.346			
	Peak No.	21	22	23	24	25	26	27	28	29	30	31	32	33	34	35	36	37	38	39	40
HQMEA	RT	44.683		47.042	49.285					58.753		65.652				75.68	77.89	79.074			94.113
	RRT	0.4748		0.4998	0.5237					0.6243		0.6976				0.8041	0.8276	0.8402			
	Area	173779		281158	7957100					68611		17283773				2106468	708442	389242			
	Area%	3.166		5.123	14.499					0.125		31.493				3.829	12.909	7.092			
	Peak No.	21	22	23	24	25	26	27	28	29	30	31	32	33	34	35	36	37	38	39	40
DHM	RT	44.722	45.949	46.853		52.981		56.444							74.05				84.556	88.296	94.131
	RRT	0.4751	0.4881	0.4977		0.5628		0.5996							0.7867				0.8983	0.9380	
	Area	494597	244318	730543		81926		253905							176592				144788	159031	
	Area%	7.612	3.76	11.243		1.261		3.907							2.718				2.228	2.447	
	Peak No.	21	22	23	24	25	26	27	28	29	30	31	32	33	34	35	36	37	38	39	40
DHM-W	RT																				94.095
	RRT																				
	Area																				
	Area%																				
	Peak No.	21	22	23	24	25	26	27	28	29	30	31	32	33	34	35	36	37	38	39	40
DHMBu	RT	44.73	45.956	46.86		53.064		56.379													94.112
	RRT	0.4753	0.4883	0.4979		0.5638		0.5991													
	Area	612916	335211	741438		70991		244478													
	Area%	8.928	4.883	10.8		1.034		3.561													
	Peak No.	21	22	23	24	25	26	27	28	29	30	31	32	33	34	35	36	37	38	39	40
DHMEA	RT	44.711	45.958	46.878		52.95		56.42							74.002				84.503	88.242	94.082
	RRT	0.4752	0.4885	0.4983		0.5628		0.5997							0.7866				0.8982	0.9379	
	Area	119330	569050	210083		296891		740681							854864				730557	709520	
	Area%	7.788	3.714	13.711		1.938		4.834							5.579				5.768	4.631	
	Peak No.	21	22	23	24	25	26	27	28	29	30	31	32	33	34	35	36	37	38	39	40
HQHLM	RT	44.676		47.054	49.268		55.643		58.011	58.965	61.993	65.537	68.403	70.852		75.407	77.594	78.766			93.967
	RRT	0.4754		0.5008	0.5243		0.5922		0.6174	0.6275	0.6597	0.6974	0.7279	0.7540		0.8025	0.8258	0.8382			
	Area	92210		117891	2150678		616583		567965	1213883	186258	963638	2244259	8447799		151326	477560	240837			
	Area%	0.324		4.146	7.564		2.169		1.998	4.269	6.551	3.389	7.893	29.711		0.532	1.68	0.847			
	Peak No.	21	22	23	24	25	26	27	28	29	30	31	32	33	34	35	36	37	38	39	40
HQHLMW	RT			47.028	49.294		55.576		57.96	58.975	61.966	65.606	68.391	70.748		75.543	77.81				94.1
	RRT			0.4998	0.5238		0.5906		0.6159	0.6267	0.6585	0.6972	0.7268	0.7518		0.8028	0.8269				
	Area			232039	4597380		735739		725173	771407	219234	202663	2372380	12429937		50954	84581				
	Area%			5.009	9.924		1.588		1.565	1.665	4.733	0.437	5.121	26.831		0.11	0.183				
	Peak No.	21	22	23	24	25	26	27	28	29	30	31	32	33	34	35	36	37	38	39	40
HQHLM-Bu	RT			46.998			55.672		58.046	58.983	62.091		68.447	71.032							93.984
	RRT			0.5001			0.5924		0.6176	0.6276	0.6607		0.7283	0.7558							
	Area			234592			687983		606641	1522742	180746		2632075	7101713							
	Area%			1.336			3.917		3.454	8.669	10.29		14.984	40.43							
	Peak No.	21	22	23	24	25	26	27	28	29	30	31	32	33	34	35	36	37	38	39	40
HQHLM-EA	RT	44.705		47.038	49.285						62.399	65.606		71.55		75.674	77.873	79.054			94.094
	RRT	0.4751		0.4999	0.5238						0.6632	0.6972		0.7604		0.8042	0.8276	0.8402			
	Area	591535		171635	5721906						135777	7771489		1948972		1067688	346559	183070			
	Area%	1.811		5.254	17.514						0.416	23.787		5.966		3.268	10.608	5.604			
DHHQM	Peak No.	21	22	23	24	25	26	27	28	29	30	31	32	33	34	35	36	37	38	39	40
	RT	44.58	45.906	46.919	49.201	52.964		56.376				65.591			73.973	75.548	77.761	78.954	84.518	88.252	94.109
	RRT	0.4737	0.4878	0.4986	0.5228	0.5628		0.5991				0.6970			0.7860	0.8028	0.8263	0.8390	0.8981	0.9378	
	Area	471055	181254	146066	1757582	63629		183744				951206			125871	128068	440079	235117	113550	126159	
	Area%	3.093	1.19	9.59	11.539	0.418		1.206				6.245			0.826	0.841	2.889	1.544	0.746	0.828	
DHHQM-W	Peak No.	21	22	23	24	25	26	27	28	29	30	31	32	33	34	35	36	37	38	39	40
	RT			46.935	49.159																94.073
	RRT			0.4989	0.5226																
	Area			364274	544765																
	Area%			6.069	9.077																
DHHQM-Bu	Peak No.	21	22	23	24	25	26	27	28	29	30	31	32	33	34	35	36	37	38	39	40
	RT	44.786	46.037	47.027	49.298	53.059		56.427				65.629				75.586	77.793	78.98	84.519	88.241	94.07
	RRT	0.4761	0.4894	0.4999	0.5241	0.5640		0.5998				0.6977				0.8035	0.8270	0.8396	0.8985	0.9380	
	Area	677120	351778	271252	3161720	71046		275772				121058				118106	198817	105193	97922	100765	
	Area%	2.7	1.403	10.816	12.607	0.283		1.1				0.483				0.471	0.793	0.419	0.39	0.402	
DHHQM-EA	Peak No.	21	22	23	24	25	26	27	28	29	30	31	32	33	34	35	36	37	38	39	40
	RT	44.458	45.803	46.761	49.045	52.733		56.014				65.303			73.899	75.481	77.693	78.884	84.481	88.223	94.066
	RRT	0.4726	0.4869	0.4971	0.5214	0.5606		0.5955				0.6942			0.7856	0.8024	0.8259	0.8386	0.8981	0.9379	
	Area	163690	588150	292524	3241396	297924		697393				5800879			676932	626604	220547	122691	568223	537502	
	Area%	4.963	1.783	8.87	9.829	0.903		2.115				17.589			2.053	1.9	6.687	3.72	1.723	1.63	
DHHLM	Peak No.	21	22	23	24	25	26	27	28	29	30	31	32	33	34	35	36	37	38	39	40
	RT	44.893	46.214	47.178		53.314	55.888	56.491	58.302	59.348			68.628	71.16	74.106				84.586	88.321	94.134
	RRT	0.4769	0.4909	0.5012		0.5664	0.5937	0.6001	0.6194	0.6305			0.7290	0.7559	0.7872				0.8986	0.9382	
	Area	415643	217226	726720		64194	579656	235334	470826	757700			1799900	6811370	144549				118377	134971	
	Area%	2.406	1.257	4.206		0.372	3.355	1.362	2.725	4.386			10.418	39.426	0.837				0.685	0.781	
	Peak No.	21		23	24		26	27			30	31	32	33	34	35	36	37	38	39	40

	Peak No.	EC50	1	2	3	4	5	6	7	8	9	10	11	12	13	14	15	16	17	18	19	20
																				Blin		
SHXXT-M	Area	16.2	230594	62813	115101	100303	185139	53368	69207	54438	436058	209226	338248	199840	651677	233221	64746	296855	241075	3863931	90547	368406
SHXXTM-W	Area	20.0	458349	126041	0	0	0	0	0	0	304584	174177	84393	0	151533	0	0	218644	0	991774	0	0
SHXXTM-Bu	Area	6.7	83957	140113	0	206954	98755	87477	93904	68337	734974	400007	802717	0	1302829	0	195089	429150	359892	8281831	206722	738291
SHXXTM-EA	Area	32.2	0	400759	0	0	599068	78876	206869	92765	0	0	331526	667462	294455	1062047	0	0	493241	614428	52441	348462
CHLM	Area	10.8	188336	100414	0	244226	0	180245	0	207009	0	0	0	0	0	0	0	481027	0	0	0	0
CHLM-W	Area	8.3	268973	103881	0	71835	0	0	0	0	0	0	0	0	0	0	0	561246	0	0	0	0
CHLM-Bu	Area	11.1	74772	55075	100197	1057469	0	890278	0	1001175	0	0	0	0	0	0	0	0	0	0	0	0
CHLM-EA	Area	104.2	52613	0	0	0	0	89376	0	232521	0	0	0	0	0	0	0	0	0	0	0	0
HQM	Area	15.8	0	0	0	0	0	0	0	0	1082084	646660	164938	264716	0	0	0	0	333670	7919887	240557	907790
HQM-W	Area	15.3	0	0	0	0	0	0	0	0	709500	440469	65630	0	0	0	0	0	171105	7136885	209635	437046
HQM-Bu	Area	5.2	0	0	0	56886	0	73517	0	0	3571275	2125948	778637	52698	66303	58991	0	0	602353	16760086	419803	2281016
HQM-EA	Area	23.3	0	0	0	0	0	0	0	0	0	0	0	1973433	0	0	0	93044	548271	2230821	222808	1535449
DHM	Area	-	265623	0	196287	0	298432	0	124724	0	76740	0	76740	160758	1152668	393115	104478	275424	210121	0	0	0
DHM-W	Area	-	459801	95599	76373	0	0	0	0	0	0	0	133874	120104	574633	0	58110	135332	0	0	0	0
DHM-Bu	Area	-	0	0	237012	0	188096	0	147782	0	0	0	1016315	164720	1655119	0	211521	509386	257344	0	0	0
DHM-EA	Area	12.8	0	0	349931	0	928600	0	286804	0	127687	0	219619	160137	775817	1771926	0	0	346364	0	0	0
HQHLM	Area	6.9	108689	52182	0	125871	0	74637	0	88067	578648	337184	84422	92917	0	0	0	244335	155471	5397723	161797	589047
HQHLM-W	Area	5.0	0	0	0	278376	0	184596	0	162973	1451430	884313	279584	0	0	0	0	60264	406472	13433326	503187	1374472
HQHLM-Bu	Area	15.9	162746	77369	0	0	0	0	0	0	333430	197369	0	0	0	0	0	332410	0	1610500	0	0
HQHLM-EA	Area	15.9	0	0	0	0	0	68315	0	127357	0	0	0	598177	0	0	0	0	409114	3568327	205444	1395916
DHHQM	Area	33.8	237077	51031	155277	0	237129	0	87673	0	588447	285574	448835	214325	804951	303350	79659	218359	319110	4151525	103266	465174
DHHQM-W	Area	24.4	280401	57812	0	0	0	0	0	0	368372	215629	116039	87058	499986	0	166966	0	86450	2927707	72481	145316
DHHQM-Bu	Area	17.0	89226	0	215038	0	200289	0	161038	0	1224855	640531	1460646	272700	1217211	0	254286	397142	590156	8068352	192236	1054036
DHHQM-EA	Area	28.8	0	0	441910	0	911382	0	290122	0	133897	0	231842	834153	808759	1660419	0	0	552479	890504	0	569537
DHHLM	Area	13.9	325681	88821	191771	137671	243726	83027	87051	58566	0	0	430732	0	906124	316968	77732	322034	130714	0	0	0
DHHLM-W	Area	15.5	512928	154386	0	69609	0	0	0	0	0	0	120790	0	144318	0	0	275075	0	0	0	0
DHHLM-Bu	Area	14.1	140300	0	385254	362981	266072	216515	213596	186899	0	0	1529142	0	3255469	69956	443695	793967	483808	0	0	0
DHHLM-EA	Area	17.4	0	0	498274	0	853501	76866	171316	66796	0	0	214729	0	310603	1551164	0	0	347814	0	0	0

	Peak No.	21	22	23	24	25	26	27	28	29	30	31	32	33	34	35	36	37	38	39	40
												Blein	Pal	Ber							Not Input
SHXXT-M	Area	357505	143628	1264238	1384335	51189	423109	151558	360092	654664	1030615	680205	1355080	5415022	98901	95601	323929	172460	85715	108594	
SHXXTM-W	Area	0	0	0	79611	0	493847	0	393859	841698	1097622	0	1586229	4938815	0	0	0	0	0	0	
SHXXTM-Bu	Area	279693	124329	1886120	1977930	0	363709	93625	348989	349837	842061	54793	1205141	6385395	0	0	0	0	0	0	
SHXXTM-EA	Area	1463117	535604	2167621	2448285	174976	0	636684	0	0	177594	4424626	91612	0	503506	606641	1892565	1029899	686894	526174	
CHLM	Area	0	0	322406	0	82637	1319034	0	1149241	2397953	3593413	0	4529627	16706289	0	0	0	0	0	0	
CHLM-W	Area	69167	0	339603	0	0	1442854	0	1211259	2636113	3714194	0	4912842	16037568	0	0	0	0	0	0	
CHLM-Bu	Area	0	0	79666	0	0	720023	0	740412	576959	2116450	0	2363654	19354611	0	74412	0	0	0	0	
CHLM-EA	Area	0	0	0	81153	0	0	0	0	0	0	0	0	0	0	0	0	0	0	0	
HQM	Area	229400	0	1710620	3453591	0	0	0	0	0	0	2274898	0	0	0	286864	960893	495479	0	0	
HQM-W	Area	0	0	905579	1515673	0	0	0	0	0	0	0	0	0	0	0	0	0	0	0	
HQM-Bu	Area	50827	0	4265655	7285245	0	0	0	0	0	0	367258	0	0	0	134231	293553	152205	0	0	
HQM-EA	Area	1737793	0	2811580	7957100	0	0	0	0	68611	0	17283773	0	0	0	2106468	7084424	3892428	0	0	
DHM	Area	494597	244318	730543	0	81926	0	253905	0	0	0	0	0	0	176592	0	0	0	144788	159031	
DHM-W	Area	0	0	0	0	0	0	0	0	0	0	0	0	0	0	0	0	0	0	0	
DHM-Bu	Area	612916	335211	741438	0	70991	0	244478	0	0	0	0	0	0	0	0	0	0	0	0	
DHM-EA	Area	1193300	569050	2100833	0	296891	0	740681	0	0	0	0	0	0	854864	0	0	0	730557	709520	
HQHLM	Area	92210	0	1178910	2150678	0	616583	0	567965	1213883	1862589	963638	2244259	8447799	0	151326	477560	240837	0	0	
HQHLM-W	Area	0	0	2320396	4597380	0	735739	0	725173	771407	2192347	202663	2372380	12429937	0	50954	84581	0	0	0	
HQHLM-Bu	Area	0	0	234592	0	0	687983	0	606641	1522742	1807463	0	2632075	7101713	0	0	0	0	0	0	
HQHLM-EA	Area	591535	0	1716351	5721906	0	0	0	0	0	135777	7771489	0	1948972	0	1067688	3465597	1830700	0	0	
DHHQM	Area	471055	181254	1460660	1757582	63629	0	183744	0	0	0	951206	0	0	125871	128068	440079	235117	113550	126159	
DHHQM-W	Area	0	0	364274	544765	0	0	0	0	0	0	0	0	0	0	0	0	0	0	0	
DHHQM-Bu	Area	677120	351778	2712521	3161720	71046	0	275772	0	0	0	121058	0	0	0	118106	198817	105193	97922	100765	
DHHQM-EA	Area	1636903	588150	2925248	3241396	297924	0	697393	0	0	0	5800879	0	0	676932	626604	2205475	1226916	568223	537502	
DHHLM	Area	415643	217226	726720	0	64194	579656	235334	470826	757700	0	0	1799900	6811370	144549	0	0	0	118377	134971	
DHHLM-W	Area	0	0	203510	0	0	887032		706097	1231620	0	0	2843007	8731318	0	0	0	0	0	0	
DHHLM-Bu	Area	1032738	613017	1423195	0	137822	395382	466293	363649	234573	0	0	1062445	8269240	0	0	0	0	0	0	
DHHLM-EA	Area	1322774	673866	2010605	0	262753	0	849432	0	0	0	0	0	0	933495	0	0	0	781181	792611	

Area%

ローディング	1	2	3	4	5	6	7	8	9	10	11	12	13	14	15	16	17	18	19	20
PC-1	0.008835	0.002802	-0.00349	0.021707	-0.01249	0.014805	-0.0039	0.01619876	-0.0315843	-0.01756	-0.01066	-0.03307	-0.00726	-0.02232	-0.00049	0.015753	-0.02007	-0.18992	-0.00653	-0.04496
PC-2	-0.01101	-0.00306	-0.0136	0.008524	-0.02503	0.004888	-0.00755	0.00207596	0.1315848	0.079228	0.015846	-0.004	-0.04013	-0.04243	-0.00238	-0.00524	0.016693	0.870239	0.026187	0.102762
PC-3	-0.00741	0.001395	-0.00599	0.007475	-0.00524	0.006545	-0.00277	0.00841961	-0.0577115	-0.03332	-0.03199	0.081232	-0.04301	-0.00329	-0.00673	-0.00368	0.010374	-0.22043	0.001917	0.042105
PC-4	-0.05349	-0.006	0.10356	0.065226	0.176793	0.048769	0.067734	0.03985875	0.02163484	0.002115	0.230882	0.018132	0.410733	0.273116	0.042998	0.034789	0.115708	-0.06627	-0.00515	0.023737
PC-5	0.023695	-0.01877	-0.02136	0.100289	-0.15805	0.073339	-0.02462	0.08372581	-0.0716108	-0.03555	0.158298	-0.01252	0.34746	-0.35856	0.067409	0.044837	-0.01343	0.046016	0.008974	0.002922
PC-6	0.057887	0.003864	0.001318	-0.12342	-0.06938	-0.13726	0.014113	-0.1674783	0.02571133	0.009621	0.322873	0.030324	0.536349	-0.23427	0.086835	0.257513	0.041997	0.011419	-0.00993	0.004926
PC-7	-0.09367	-0.00421	0.0117	0.002873	0.071636	-0.01899	0.027668	-0.0197881	-0.3756751	-0.23048	-0.04766	0.059596	0.152943	0.099512	0.028831	0.017028	0.015927	0.334349	0.029673	-0.19392

ローディング	21	22	23	24	25	26	27	28	29	30	31	32	33	34	35	36	37	38	39
PC-1	-0.04148	-0.00861	-0.09624	-0.19262	-0.00279	0.059762	-0.0099	0.053144	0.091723	0.139688	-0.25815	0.200396	0.866586	-0.01046	-0.03189	-0.10834	-0.05923	-0.01042	-0.00956
PC-2	-0.03691	-0.01995	0.124667	0.324343	-0.00821	0.012848	-0.02393	0.013972	0.013042	0.060133	0.016477	0.043314	0.268237	-0.0217	0.005723	0.01229	0.005069	-0.01904	-0.01813
PC-3	0.061709	-0.00694	0.046931	0.278692	-0.00102	0.016048	-0.00616	0.014503	0.028649	0.0502	0.785866	0.055391	0.287163	-0.00324	0.096183	0.32267	0.176523	-0.00207	-0.00336
PC-4	0.308234	0.168855	0.509398	0.066641	0.058805	-0.05265	0.175784	-0.0366	-0.18218	-0.18894	-0.0704	-0.21703	0.177083	0.129387	-0.00732	-0.03732	-0.01818	0.118602	0.112586
PC-5	-0.13945	-0.06129	-0.31786	-0.04031	-0.04995	-0.09551	-0.11416	-0.07086	-0.30172	-0.39446	0.059851	-0.38253	0.153073	-0.1812	0.015918	0.035108	0.017099	-0.16425	-0.15381
PC-6	0.04332	0.024479	0.064883	-0.04325	-0.01303	0.131038	-0.02812	0.09002	0.322799	0.108651	0.044591	0.438636	-0.14482	-0.1274	-0.00613	0.019021	0.015151	-0.10845	-0.10185
PC-7	0.145264	0.051621	-0.13496	-0.55526	0.016715	-0.05187	0.064942	-0.01787	-0.08818	0.384591	0.236584	-0.13759	-0.01437	0.059679	-0.00227	0.039221	0.02286	0.051958	0.049783

-20000000	0
20000000	0
0	15000000
0	-10000000

-0.4	0
1	0
0	-0.2
0	1

スコア

	PC-1	PC-2	PC-3	PC-4	PC-5	PC-6	PC-7
SHXXT-M	1223758	822100.9	-670358	-18792	-91400.6	212012.2	280945.4
SHXXTM-W	2098075	-2393009	-1267563	-1330403	-56332	96162.16	-83026.7
SHXXTM-Bu	1154769	5263878	-1900463	665804	683407.1	524512.5	945986.3
SHXXTM-EA	-4922871	-3229232	2480250	856896.6	-984625	-379057	58778.51
CHLM	1.37E+07	189014.1	2718554	-513711	-1093722	488625.2	-59472.5
CHLM-W	1.32E+07	35234.3	2563581	-794921	-1531782	976584.2	-77428.2
CHLM-Bu	1.51E+07	665039.3	3232435	1072959	1615515	-2184970	-161200
CHLM-EA	-2580231	-4780105	-2608695	-1454154	481854.2	-636193	-280157
HQM	-5748670	3764147	-1135080	-903095	212654.5	-286012	189055.5
HQM-W	-4359996	2191079	-3778895	-1320169	381879.9	-459208	756087.9
HQM-Bu	-7986115	1.36E+07	-3867736	488156	-584449	-4238.93	-1396220
HQM-EA	#######	570724.6	1.63E+07	-561792	444613.1	440014.2	262797.7
DHM	-2698881	-4819502	-2622699	-88346.5	261076	71078.63	7821.152
DHM-W	-2573558	-4835430	-2657330	-1211323	694674.9	-162444	-174106
DHM-Bu	-2701732	-4808980	-2658518	241832.2	813916.1	861243.7	49416.68
DHM-EA	-2935967	-4776810	-2521473	1607359	-1402566	-632732	192168
HQHLM	3713994	3432572	489654.1	-680551	-455020	90878.91	226048.6
HQHLM-W	5287908	1.27E+07	-212353	387885.5	-85884.5	-497153	670331.8
HQHLM-Bu	4241503	-1155460	-666260	-1438269	-757281	513288	156158.4
HQHLM-EA	-5444964	1217383	6623130	-567918	651602.6	-478986	-537763
DHHQM	-4250066	-347728	-2079721	-73165.4	192158	119444.6	129003.3
DHHQM-W	-3295529	-1975266	-3152462	-1195327	628436.5	-196725	246658.8
DHHQM-Bu	-5212196	3836540	-3301996	963674.8	208592.8	881717.8	-100992
DHHQM-EA	-5701041	-2658070	3879077	1779969	-1227562	-234200	25581.65
DHHLM	3715151	-2875124	-541434	441763.2	369228.7	210973.3	-516507
DHHLM-W	5757196	-2293956	85397.23	-650427	229401.2	196461.1	-962419
DHHLM-Bu	4654084	-2531684	-248097	2871186	1911738	1355530	-43999.2
DHHLM-EA	-2915934	-4788354	-2503560	1404321	-1493784	-882430	182815

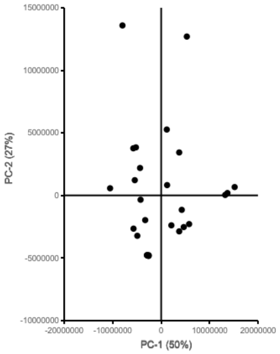

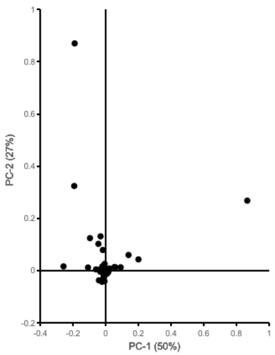

Supplement: Supplementary file 2 — Supplementary file2 (PDF 33173 kb) [file 11418_2019_1382_MOESM2_ESM.pdf]
